# Structures and Biological Activities of Secondary Metabolites from *Daldinia* spp.

**DOI:** 10.3390/jof10120833

**Published:** 2024-12-02

**Authors:** Miao Yu, Shiji Chen, Jueying Shi, Weikang Chen, Yikang Qiu, Jing Lan, Shiyan Qu, Jiayi Feng, Ru Wang, Fangru Lin, Guolei Huang, Caijuan Zheng

**Affiliations:** 1 Key Laboratory of Tropical Medicinal Resource Chemistry of Ministry of Education, College of Chemistry and Chemical Engineering, Hainan Normal University, Haikou 571158, China; yumiaonpc@126.com (M.Y.); chenshijinpc@126.com (S.C.); jueying202406@163.com (J.S.); 18971192012@163.com (W.C.); qyk7747226@sina.com (Y.Q.); seawry@163.com (J.L.); 15297508701@163.com (S.Q.); 19308057394@163.com (J.F.); 15035429959@163.com (R.W.); fl326688@163.com (F.L.); 2 Key Laboratory of Tropical Medicinal Plant Chemistry of Hainan Province, Haikou 571158, China

**Keywords:** *Daldinia* sp., secondary metabolites, bioactivity

## Abstract

The genus *Daldinia* have long been recognized as a source of structural novel, pharmaceutically relevant natural products. We reviewed the structures and activities of secondary metabolites isolated from the genus of *Daldinia* from January 1995 to June 2024, and 280 compounds, including six major categories—terpenoids, alkaloids, polyketides, polyphenols, steroids, and other classes—are presented in this review. Among these metabolites, 196 were identified as new structures. Remarkably, 112 compounds exhibited a range of biological activities, including cytotoxic, antimicrobial, anti-inflammatory, antifungal, anti-virus, and enzyme-inhibitory activities. This review highlights the bioactive metabolites discovered in the past three decades from the genus of *Daldinia* while also exploring the potential of these symbiotic fungi as rich sources of novel and diverse natural products. The varying bioactivities of these metabolites offer a vast array of promising lead compounds and also could significantly contribute to the development of new medicines.

## 1. Introduction

Endophytes are microorganisms that inhabit diverse organs, tissues or intercellular spaces of plants, animals and other resources, while the hosts generally do not show any symptoms of infection. In general, endophytes include endophytic fungi, endophytic bacteria and endophytic actinomycetes. Endophytes, as a highly significant microbial resource, are prevalent in nature and omnipresent within a diverse range of terrestrial and aquatic plants. Endophytes have been successfully isolated from a diverse range of plants, animals and other resources, including bryophytes, ferns, pteridophytes, hornworts, herbaceous plants, various types of woody plants and some animals [1,2]. Endophytic fungi have gained attention owing to their potential for producing novel bioactive secondary metabolites, with diverse structures and potent activities. The metabolites produced by fungi through various biosynthetic pathways, such as steroids, terpenoids, peptides, alkaloids and polyketides, have shown potent bioactivities, including properties like anticancer, antiviral, antibacterial and antifungal activities, among others [3,4,5]. Thus, endophytic fungi represented a valuable source of novel compounds characterized by structural diversity and pharmacological properties.

Recent advances in fungal genome sequencing and bioinformatics analysis indicate that the secondary metabolite biosynthetic potential of fungi is underappreciated as a greater number of secondary metabolite biosynthetic gene clusters are present in the genome. This is because many of the secondary metabolite gene clusters are silent or lowly expressed under laboratory conditions [6,7]. Accordingly, great efforts have been made to activate the silent/cryptic biosynthetic gene clusters, leading to the discovery of novel secondary metabolites. Therefore, the activation of these silent biosynthetic gene clusters is essential to current natural product discovery research. Several successful strategies have been developed, including chemical epigenetic modification, co-cultivation and the One Strain Many Compounds (OSMACs) approach. These methods are simple and effective for regulating fungal metabolism to activate silent metabolic pathways in the fungus, resulting in the production of cryptic natural products. With the rapid advancement of sequencing technologies, an increasing amount of genomic information pertaining to fungi has been elucidated. Subsequently, molecular biology techniques, such as genome mining, heterologous expression and genome mining in conjunction with the OSMAC approach, were utilized to modulate fungal metabolism [8,9,10,11,12].

**Figure 1:**
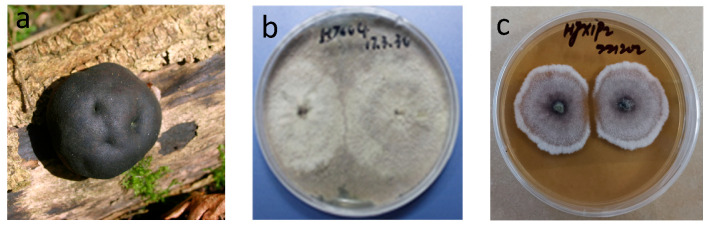
The images of (**a**–**c**) of *Daldinia* spp. (**a**): *Daldinia concentrica*; (**b**,**c**): *Daldinia eschscholtzii*.

As a significant microbial resource, endophytic fungi are widely distributed in nature. Among fungi, the genus *Daldinia* sp. represented a prevalent endophytic fungus found in both terrestrial and marine environments (Figure 1). The genus *Daldinia* was described by Cesati and De Notaris (1863) and belongs to Xylariales. Xylariales is one of the largest families in this order, and both the family and the *Daldinia* genus have been studied exhaustively for secondary metabolite production [13].

Therefore, this review updates current compounds to cover metabolites isolated from the fungi genus *Daldinia* between January 1995 and June 2024. It also provides the structural diversity of the compounds, as well as detailed information on their sources and associated bioactivity. Two hundred and eighty compounds were isolated from the genus *Daldinia*. Among these isolated compounds, 196 compounds were new compounds. Remarkably, 112 compounds showed various biological activities, such as cytotoxic, antimicrobial, anti-inflammatory, antifungal, antivirus and enzyme-inhibitory activities. Chemical epigenetic modification, co-cultivation, the OSMAC approach and genome mining were employed to activate the silent/cryptic biosynthetic gene clusters within the genus *Daldinia*, with the aim of obtaining structurally novel and bioactive secondary metabolites. Liquid chromatography–tandem mass spectrometry (LC-MS/MS)-based molecular networking has become a highly efficient strategy for the discovery of new natural products from complex mixtures. The structures and absolute configurations of the new compounds and novel skeleton compounds were elucidated by the analysis of nuclear magnetic resonance (NMR) spectroscopy, MS data, electronic circular dichroism (ECD) calculations and single-crystal X-ray diffraction. This study contributes to a better understanding of the chemical structures and biological activities of secondary metabolites from *Daldinia* spp., thereby enhancing the process of drug discovery and development.

## 2. Secondary Metabolites from *Daldinia* spp. and Their Bioactivities

### 2.1. Terpenoids

**Figure 2:**
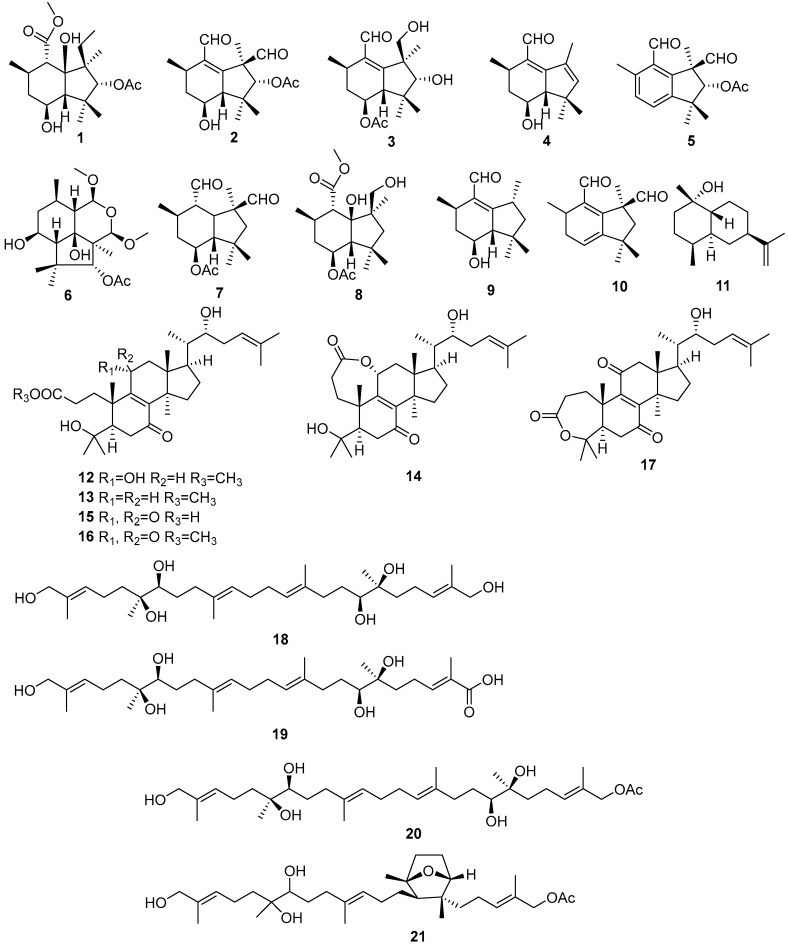
Terpenoids produced by *Daldinia* species (**1**–**21**).

Terpenoids are relatively rare, isolated from the genus *Daldinia*. Only 21 terpenoids were obtained from the fungi genus *Daldinia* in the last thirty years. Here, 21 terpenoids, with 16 new terpenoids (including 6 sesquiterpenes, 6 diterpenes and 4 triterpenes), were isolated and identified from the *Daldinia* spp. (Figure 2).

Six new sesquiterpenes, methyl-7*α*-acetoxydeacetylbotryoloate (**1**), 7*α*-acetoxydeacetylbotryenedial (**2**), 7*α*-hydroxybotryenalol (**3**), 7,8-dehydronorbotryal (**4**), 7*α*-acetoxydehydrobotrydienal (**5**), 7*α*-acetoxy-15-methoxy-10-*O*-methyldeacetyldihydrobotrydial (**6**), together with four known compounds, 7*α*-hydroxy-10-*O*-methyldihydrobotrydial (**7**), 7-hydroxy-16-*O*-methyldeacetyldihydrobotrydial-hydrate (**8**), 7-hydroxydeacetyl-botryenalol (**9**), and 7*α*-hydroxydihydrobotrydial (**10**), were isolated from the fungus *Daldinia concentrica* (S0318) collected at Laojunshan, Yunnan Province, China [14]. One known compound, rel-(1*S*,4*S*,5*R*,7*R*,10*R*)-10-desmethyl-1-methyl-11-eudesmene (**11**), was isolated from the lichen *Punctelia* sp. derived fungus *Daldinia childiae*; the fungus was collected from Cloak Mountain Scenic Area in Guizhou Province of China. Compound **11** exhibited slight *α*-amylase inhibitory activity at the concentration of 1.5 mg/mL, with an inhibition rate reaching up to 15.99% [15]. Six new 3,4-secolanostane triterpenoids, daldiconoids B–G (**12**–**17**), were isolated from the fungus *Daldinia concentrica* collected from Yunnan Province, China. Compounds **12** and **14**–**16** inhibited the expressions of IL-1*β*, IL-6 and TNF-*α* in lipopolysaccharide-stimulated RAW264.7 cells comparable to the positive control dexamethasone at a concentration of 10 μM. Compound **12** blocked the JAK2/STAT3 signaling pathway induced by lipopolysaccharide [16]. One new triterpenoid, concentricol (**18**), was isolated from the fungus *Daldinia concentrica* [17]. Three new triterpenoids, concentricols B–D (**19**–**21**), were isolated from the white wax tree trunk-derived fungus *Daldinia concentrica* (North Rhine Westphalia, Germany) [18] (Figure 2).

### 2.2. Alkaloids

Alkaloids are an important class of natural products containing a nitrogen heterocycle moiety, which are found in a diverse array of natural sources and exhibit a variety of biological activities. There were 53 alkaloids (including 43 new compounds) isolated and identified from *Daldinia* spp., including cytochalasins, indole alkaloids, etc. Among them, 21 compounds showed biological activities such as antiviral, antibacterial and cytotoxic properties.

#### 2.2.1. Cytochalasins

**Figure 3:**
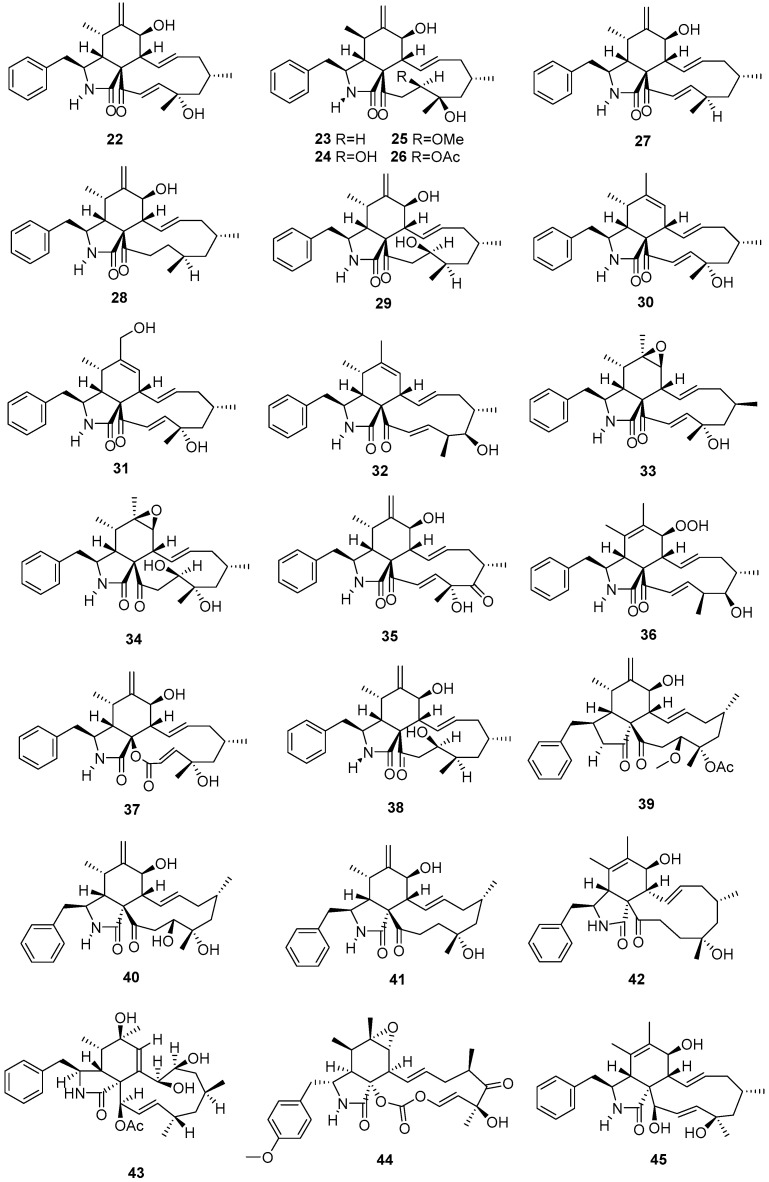
Cytochalasins produced by *Daldinia* species (**22**–**45**).

Five new cytochalasins, (11)-cytochalasa-6(12), 13,19-triene-1,21-dione-7,18-dihydroxy-16,18-dimethyl-10-phenyl-(7S*,13*E*,16S*,18S*,19*E*) (**22**), (11)-cytochalasa-6(12),13-diene-1,21-dione-7,18-dihydroxy-16,18-dimethyl-10-phenyl-(7S*,13*E*,16S*,18R*) (**23**), (11)-cytochalasa-6(12),13-diene-1,21-dione-7,18,19-trihydroxy-16,18-dimethyl-10-phenyl-(7S*,13*E*,16S*,18S*,19R*) (**24**), (11)-cytochalasa-6(12),13-diene-1,21-dione-7,18-dihydroxy-16,18-dimethyl-19-methoxy-10-phenyl-(7S*,13*E*,16S*,18S*,19R*) (**25**), and (11)-cytochalasa-6(12),13-diene-1,21-dione-7,18-dihydroxy-16,18-dimethyl-19-acetoxy-10-phenyl-(7S*,13*E*,16S*,18S*,19R*) (**26**), were isolated from the fungus *Daldinia* sp. from *Quercus acutissima* in Tokushima [19]. Eleven new cytochalasins, (11)-cytochalasa-6(12),13,19-triene-l,21-dione-16,18-dimethyl-7-Hydroxy-10-phenyl-(7S*,3*E*,16S*,18R*,19*E*) (**27**), (11)-cytochalasa-6(12),13-diene-1,21-dione-16,18-dimethyl-7-hydroxy-10-phenyl-(7S*,13*E*,16S*,18S*) (**28**), (11)-cytochalasa-6(12),13-diene-1,21-dione-7,19-dihydroxy-16,18-dimethyl-10-phenyl-(7S*,13*E*,16S*,18R*,19R*) (**29**), (11)-cytochalasa-6,13,19-triene-l,21-dione-18-hydroxy-16,18-dimethyl-10-phenyl-(6*Z*,13*E*,16S*,18S*,19*E*) (**30**), (11)-cytochalasa-6,13,19-triene-1,21-dione-12,18-dihydroxy-16,18-dimethyl-10-phenyl-(6*E*,13*E*,16S*,18S*,19*E*) (**31**), (11)-cytochalasa-6,13,19-triene-l,21-dione-17-hydroxy-16,18-dimethyl-10-phenyl(6*Z*,13*E*,16S*,17*R*,18S*,19*E*) (**32**), (11)-cytochalasa-13,19-diene-1,21-dione-6,7-epoxy-18-hydroxy-16,18-dimethyl-10-phenyl-(7S*,13*E*,16S*,18S*,19*E*) (**33**), (11)-cytochalas-13-ene-1,21-dione-6,7-epoxy-18,19-dihydroxy-16,18-dimethyl-l0-phenyl-(7S*,13*E*,16S*,18S*,19R*) (**34**), (11)-cytochalasa-6(12),13,19-triene-1,17,21-trione-7,18-dihydroxy-16,18-dimethyl-10-phenyl-(7S*,13*E*,16S*,l8R*,19*E*) (**35**), (11)-cytochalasa-5,13-19-triene-1,21-dione-7-hydroperoxy-17-hydroxy-16,18-dimethyl-10-phenyl-(5Z*,7S*,13*E*, 16S*,17R*,18S*,19*E*) (**36**), and 22-oxa-(12)-cytochalasa-6(12),13,19-triene-l,21-dione-7,18-dihydroxy-16,18-dimethyl-10-phenyl-(7S*,13*E*,16S*,18S*,19*E*) (**37**), were isolated from an unidentified fungus *Daldinia* sp. from the *Quercus acutissima* in Tokushima [20]. A new cytochalasin, [11]-cytochalasa-6(12),13-diene-1,21-dione-7,19-dihydroxy-16,18-dimethyl-10-phenyl-(7S*,13*E*,16S*,18R*,19R*) (**38**), was also isolated from the *Quercus acutissima*-derived fungus *Daldinia vernicosa* collected in Tokushima [21]. One new cytochalasin daldinin (**39**), and two known cytochalasins [11]-cytochalasa-6(12),13-diene-1,21-dione-7,18,19-trihydroxy-16,18-dimethyl-10-phenyl-(7S*,13*E*,16S*,18S*,19R*) (**40**), and [11]-cytochalasa-6(12),13-diene-1,21-dione-7,18-dihydroxy-16,18-dimethyl-10-phenyl-(7S*,13*E*,16S*,18R*) (**41**), were isolated from the fungus *Daldinia concentrica* (Pumat National Park of Nghe An Province, Vietnam). Compound **39** showed cytotoxicity against five human tumor cells SK-LU-1, HepG2, Hep3B, SW480 and MCF7, with IC_50_ values of 11.4 ± 0.5, 13.5 ± 1.3, 13.3 ± 1.4, 13.1 ± 0.9 and 13.5 ± 1.2 µM, respectively. Compounds **40** and **41** showed weak cytotoxicity against all the tested tumor cell lines, with the IC_50_ values ranging from 23.0 ± 1.1 to 58.2 ± 2.3 µM, respectively [22]. One new cytochalasin, [11]-cytochalasa-5(6),13-diene-1,21-dione-7,18-dihydroxy-16,18-dimethyl-10-phenyl-(7S*,13*E*,16S*,18R*) (**42**), was isolated from the mangrove *Brguiera sexangula var. rhynchopetala*-derived fungus *Daldinia eschscholtzii* HJ001 (South China Sea, China). Compound **42** exhibited weak antibacterial activity against *Escherichia coli*, *Staphylococcus aureus*, *Bacillus cereus*, *Vibrio parahaemolyticus* and *Vibrio alginolyticus* with the same MIC value of 50 μg/mL [23]. One new cytochalasin, 21-(acetyloxy)-6,13,14-trihydroxy-16,18-dimethyl-10-phenyl [11] cytochalasa-7,19-dien-1-one (**43**), was isolated from the fungus *Daldinia concentrica*, which was collected from Laojunshan, Yunnan [24]. One known compound, phenochalasin B (**44**), was isolated from the European ash-derived fungus *Daldinia concentrica* (North Rhine Westphalia, Germany) [25]. One known compound, cytochalasin O (**45**), was isolated from a mangrove plant *Ceriops tagal*-derived fungus *Daldinia eschscholzii* MCZ-18 (Dong Zhai Gang-Mangrove Garden on Hainan Island, China) [26] (Figure 3).

#### 2.2.2. Indole Alkaloids

**Figure 4:**
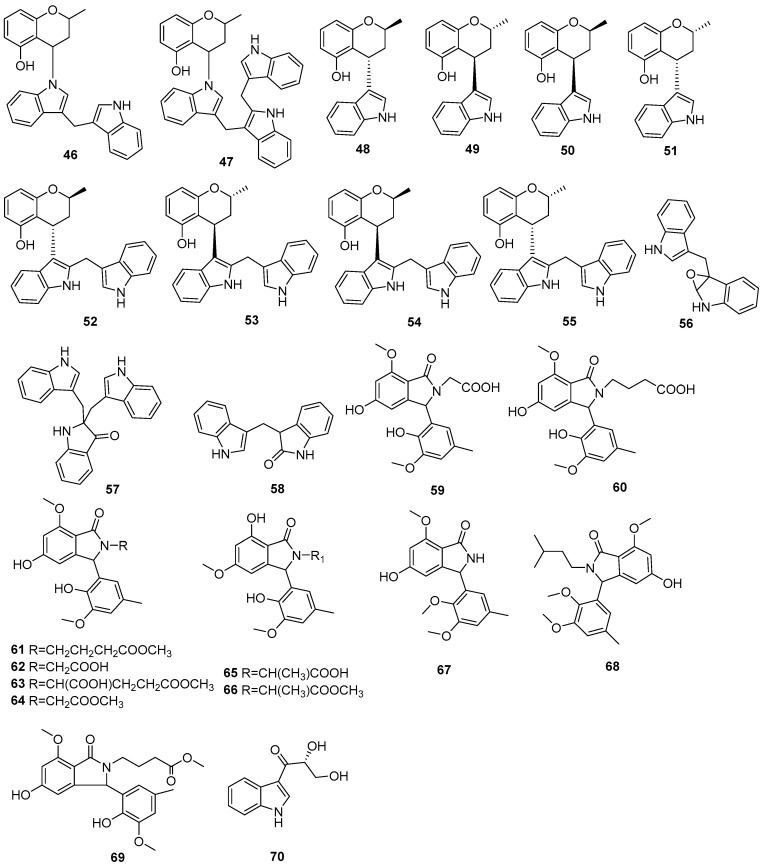
Indole alkaloids produced by *Daldinia* species (**46**–**70**).

**Scheme 1:**
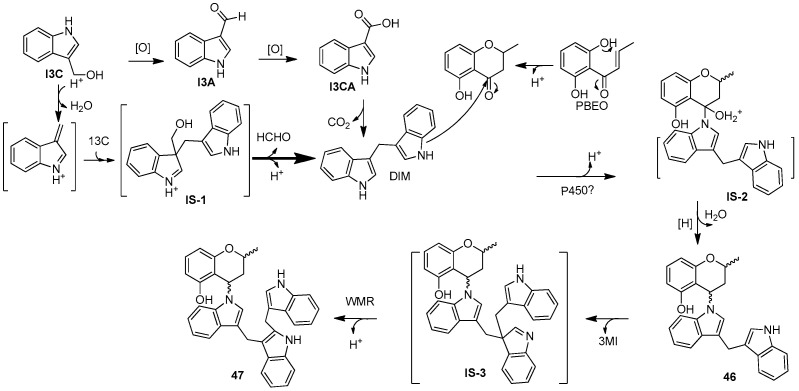
The putative biosynthetic pathways of **46** and **47** [27].

**Scheme 2:**
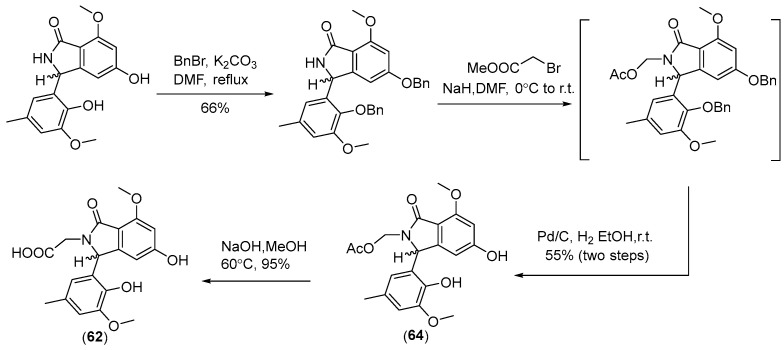
Synthesis of **62** and **64** [32].

**Scheme 3:**
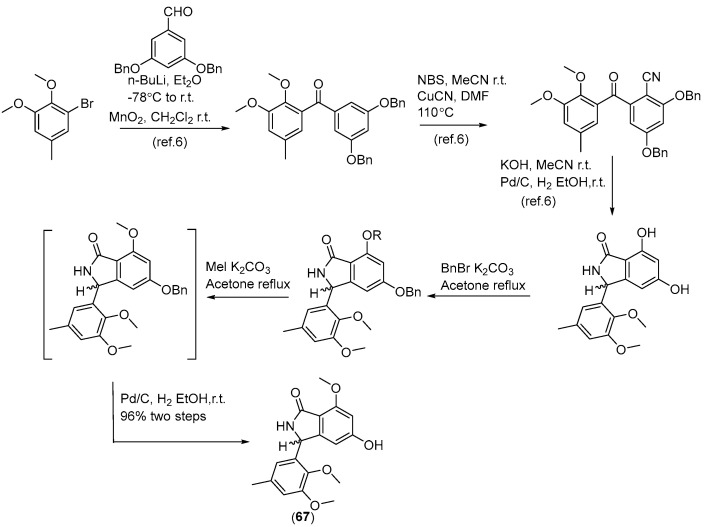
Synthesis of **67** [32].

Two new skeletally undescribed polyketide–indole hybrid alkaloids, indolpolyketones A (**46**) and B (**47**), were isolated from the fungus *Daldinia escosholzii* from the *Tenodera aridifolia* gut. Compounds **46** and **47** were isolated and assigned to be constructed through C-N bond formation, which were reported for the first time and were the first example of polyketide–indole hybrids pieced together through C-N bond formation in the ^13^C-exposed culture of *D. eschscholzii*. This expands the knowledge about the chemical production of *D. eschscholzii* through precursor-directed biosynthesis. Therefore, compound **46** was hypothesized to be produced through the Michael addition of DIM with PBEO, which may be catalyzed by an uncertain P450 enzyme. Compound **47** was generated through another round of Michael addition of **46** with 3-methyleneindolium and followed by Wagner–Meerwein rearrangement (Scheme 1). Compounds **46** and **47** showed significant antiviral activity against H1N1 with IC_50_ values of 45.2 and 31.4 μM, respectively [27]. Two pairs of new skeletally undescribed polyketide–indole hybrids (PIHs) were generated from indole-3-carbinol in the fungus *Daldinia eschscholzii* from the gut of *Tenodera aridifolia*. Each indolchromin alkaloid was chirally separated into four isomers, (2*S*,4*R*)-indolchromin A (**48**), (2*R*,4*S*)-indolchromin A (**49**), (2*S*,4*S*)-indolchromin A (**50**), (2*R*,4*R*)-indolchromin A (**51**); and (2*S*,4*R*)-indolchromin B (**52**), (2*R*,4*S*)-indolchromin B (**53**), (2*S*,4*S*)-indolchromin B (**54**), (2*R*,4*R*)-indolchromin B (**55**). Furthermore, the indolchromin construction pathways in fungal culture were clarified through enzyme inhibition, a precursor feeding experiment and energy calculation. The cascade reactions, including decarboxylative Claisen condensation catalyzed by 8-amino-7-oxononanoate synthase (AONS), C(*sp*^3^)-H activation, double-bond migration and Michael addition, were compatible during fungal cultivation. Compound **48** was inhibitory against *Clostridium perfringens*, *C. difficile*, *Veillonella* sp., *Bacteroides fragilis* and *S. pyogenes*, with MIC values of 2.5, 2.5, 5.0, 6.4 and 2.1 μM, respectively. Compound **49** was inhibitory against *C. perfringens*, *C. difficile*, *Veillonella* sp., *Bacteroides fragilis* and *S. pyogenes*, with MIC values of 1.3, 2.5, 5.0, 6.2 and 5.0 μM, respectively. Compound **53** was inhibitory against *C. perfringens*, *C. difficile* and *S. pyogenes*, with MIC values of 6.7, 6.3 and 6.2 μM, respectively. Compounds **49** and **54** showed cytotoxic activity against the human breast cancer cell lines MDA-MB-231 with IC_50_ values of 27.9 and 131.2 nM, respectively, and **49** was additionally active against human breast cancer cell line MCF-7, with an IC_50_ value of 94.4 nM [28]. Two new alkaloids, dalesindoloids A (**56**) and B (**57**), together with one known compound 3-(1*H*-indole-3ylmethyl)-2-oxindole (**58**), were isolated from the gut of *Tenodera aridifolia*-derived fungus *D*. *eschscholzii*. Compounds **56** and **57** exhibited cytotoxic activity against the leukemia HL-60 cell line with IC_50_ values of 1.0 and 7.4 μM, respectively, and compound **56** also showed antibacterial activity against *S. aureus*, with an MIC value of 9.1 μM [29]. Two new alkaloids, daldinans B (**59**) and C (**60**), were isolated from the fungus *Daldinia concentrica* collected in Yamanashi Prefecture, Japan [30]. Four new isoindolinone derivatives, daldinans D–G (**63**–**66**), together with two known compounds, daldinans A (**61**) and B (**62**), were isolated from the stroma of the ascomycete *Daldinia concentrica* collected at Odaesan National Park in Korea. Compounds **63**–**66** exhibited potent antioxidant activities with IC_50_ values of 2.65, 3.40, 3.50 and 3.21 μM, respectively. Compounds **61** and **62** exhibited moderate antioxidant activities, with IC_50_ values of 12.62 and 39.67 μM, respectively. The structure–activity relationship study indicated that **61** and **64** had a methoxy group at C-7, suggesting the methylation of the carboxyl group in the alkyl side chain enhanced the free radical scavenging activities, and compounds **65** and **66**—with a methoxy group at C-5—exhibited similar activity. The methoxy group at C-5 and the methylation of the carboxyl group in the alkyl side chain were crucial to enhancing free-radical scavenging activity [31]. Three known N-alkylated 3-phenyl isoindolinones, entonalactam B (**67**), daldinan B (**62**) and daldinan E (**64**), were isolated from *Daldinia* sp. Compounds **62**, **64** and **67** were synthesized, and the conformational properties of these isoindolinones were examined using their derivatives, revealing the presence of atropisomers (Scheme 2 and Scheme 3) [32]. A pair of new racemic (±) childinins C (**68**) were isolated from the fungus *Daldinia childiae* from Kunming Botanical Garden in China [33]. One new alkaloid, daldinin A (**69**), was isolated from the fungus *Daldinia concentrica* (Muju county, Jeonbuk province, Korea). Compound **69** exhibited potent 2,2′-azinobis(3-ethylbenzothiazoline-6-sulfonate) radical scavenging activity with an IC_50_ value of 10.4 μM, which was stronger than the positive control butylated hydroxyanisole (IC_50_ = 10.8 μM) [34]. One new alkaloid, 1-(3-indolyl)-2*R*,3-dihydroxypropan-1-one (**70**), was isolated from the marine-associated fungus *Daldinia eschschilzii* collected from *Scaevola serica* branches (Hainan, China) [35] (Figure 4).

#### 2.2.3. Other Alkaloids

**Figure 5:**
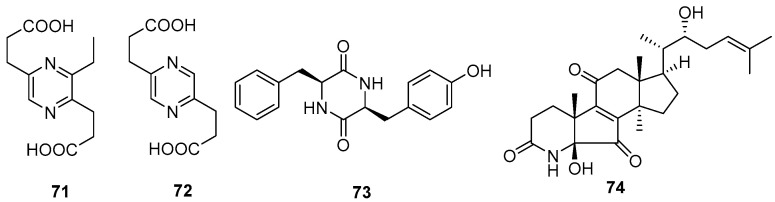
Other alkaloids produced by *Daldinia* species (**71**–**74**).

**Scheme 4:**
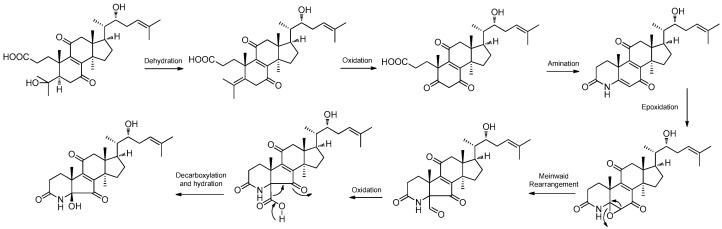
Putative biogenetic pathway for **74** [16].

One new alkaloid, 3-ethyl-2,5-pyrazinedipropanoic acid (**71**), along with two known compounds, 2,5-pyrazinedipropanoic acid (**72**) and cyclo-(Phe-Tyr) (**73**), were isolated from the marine-associated fungus *Daldinia eschschilzii* (Hainan, China) [35]. One undescribed alkaloid, daldiconoid A (**74**), was isolated from the fungus *Daldinia concentrica* (Yunnan Province, China). Compound **74** was a highly modified 4,6,28,29-tetranorlanostane triterpenoid alkaloid featuring an unusual *δ*-lactam fused with a flanking cyclopentenone architecture. A plausible biosynthetic pathway for **74** was proposed. The co-isolate 4,22*R*-dihydroxy-3,4-secolanosta-8,24-dien-7,11-dione-3-oic acid was found in relatively high abundance (0.003%) and was considered the biosynthetic precursor. Dehydration between OH-4 and H-5 in 4,22*R*-dihydroxy-3,4-secolanosta-8,24-dien-7,11-dione-3-oic acid generated a double bond Δ4, which was subsequently oxidized to a ketone, then the amination, epoxidation, Meinwald rearrangement, oxidized, decarboxylation and nucleophilic addition reactions were used to biosynthesize **74** (Scheme 4). Compound **74** inhibited the expressions of IL-1*β*, IL-6 and TNF-*α* in lipopolysaccharide-stimulated RAW264.7 cells at a concentration of 10 μM. Mechanistically, **74** blocked the JAK2/STAT3 signaling pathway induced by lipopolysaccharide [16] (Figure 5).

### 2.3. Polyketides

Polyketides are one of the most diverse natural product categories. Here, 125 polyketides were isolated and identified from the fungi genus Daldinia, including 85 new compounds, and 54 compounds exhibited biological activities such as cytotoxicity, antioxidant, insect resistance activity and antibacterial properties.

#### 2.3.1. Xanthones

**Scheme 5:**
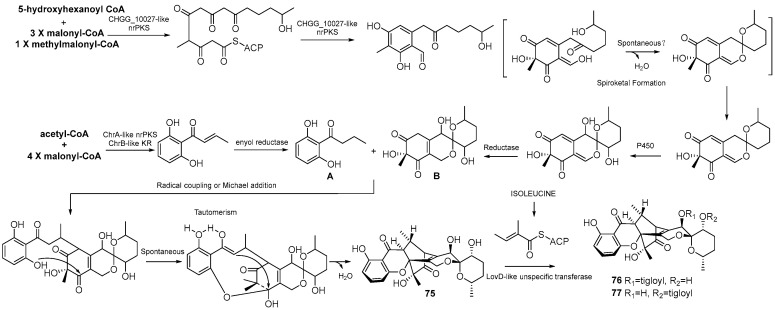
Putative biosynthetic pathway of compounds **75**–**77** [36].

**Scheme 6:**
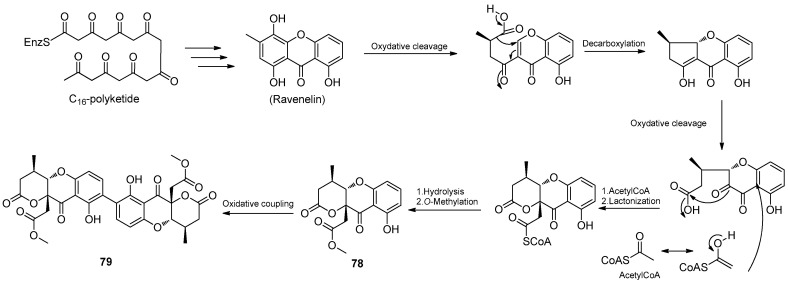
Putative biosynthetic pathway of compounds **78**–**79** [37].

**Figure 6:**
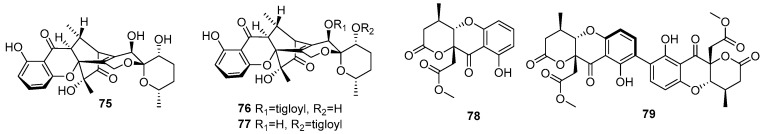
Xanthones produced by *Daldinia* species (**75**–**79**).

Three new compounds, daldipyrenones A–C (**75**–**77**), were isolated from the lichen *Myelochroa aurulenta*-derived fungus *Daldinia pyrenaica* 047188 (Korea). Compound **75** exhibited significant antimelanogenic activity with an EC_50_ value of 3.36 μM, better than positive controls arbutin (54.49 μM) and hydroquinone (4.66 μM), and showed moderate adiponectin-secretion promoting activity. Daldipyrenones are likely derived by combining a chromane biosynthesis intermediate, 1-(2,6-dihydroxyphenyl)but-2-en-2-one, and a spiro-azaphilone, pestafolide A, via a radical coupling or Michael addition to form a bicyclo[2.2.2]octane ring. Genome sequencing of the strain revealed two separate biosynthetic gene clusters responsible for forming two biosynthetic intermediates, suggesting a proposed biosynthetic pathway (Scheme 5) [36]. Two new compounds, daldiniaeschsones A (**78**) and B (**79**), were isolated from the *Cinnamomum bejolghota*-derived fungus *Daldinia eschscholtzii* SDBRCMUNKC745 (Chiang Mai, Thailand). The putative biosynthetic pathway of compounds **78** and **79** are shown in Scheme 6. Compounds **78** and **79** showed potent *α*-glucosidase inhibitory activity, with IC_50_ values of 0.16 and 0.23 mM, respectively [37] (Figure 6).

#### 2.3.2. Chromones

**Figure 7:**
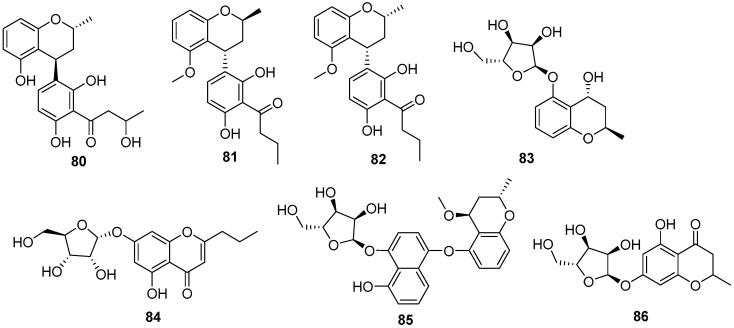

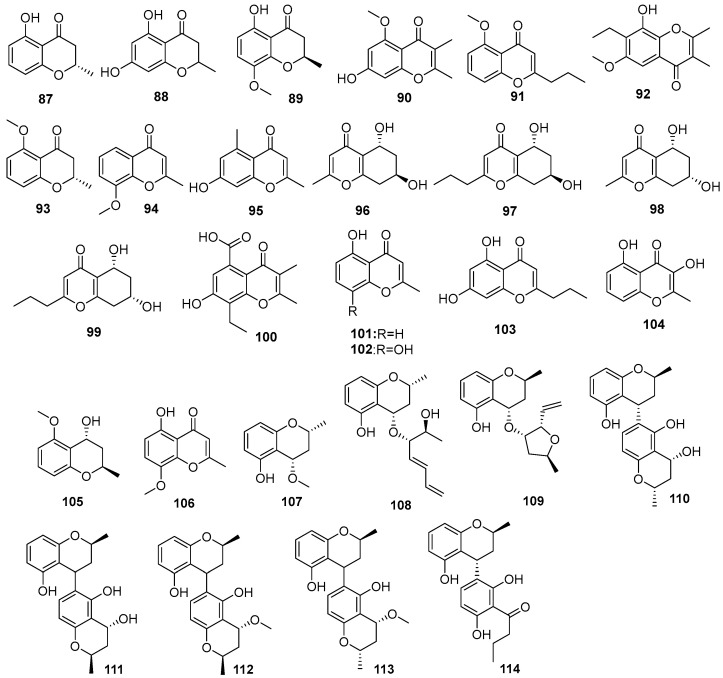
Chromones produced by *Daldinia* species (**80**–**114**).

One new polyketide, nodulisporin G (**80**), was isolated from the *Tenodera aridifolia*-derived fungus *Daldinia eschscholzii*, the fungus was collected from Purple Mountain in the suburb of Nanjing, China [38]. Two new compounds, 8-*O*-methylnodulisporin F (**81**) and nodulisporin H (**82**), were obtained from the mangrove *Brguiera sexangula* var. *rhynchopetala*-derived fungus *Daldinia eschscholtzii* HJ004 (South China Sea, China). Compounds **81** and **82** showed moderate antibacterial activity against *Staphylococcus aureus*, methicillin-resistant *S. aureus* (MRSA) and *Bacillus cereus*, **81** with MIC values of 6.25, 12.5 and 6.25 µg/mL, respectively, and **82** with MIC values of 12.5, 12.5 and 6.25 µg/mL, respectively [39]. One new compound, daldiniside A (**83**), was isolated from the *Scaevola serica*-derived fungus *D. eschschilzii* (Hainan, China) [35]. One known compound, 7-*O*-*α*-*D*-ribosyl-5-hydroxy-2-propylchromone (**84**), was isolated from *Euphorbia pekinensis*-derived fungus *Daldinia eschscholtzii* J11 (Xishuang Banna Tropical Botanical Garden, Yunnan Province, China) [40]. OSMAC strategy was used to isolate compounds from the *Dendrobium officinale*-derived fungus *Daldinia eschscholzii* JC-15 (Yunnan, China). Daldinsin (**85**), with an unprecedented benzopyran–naphthalene hybrid skeleton, was isolated from red ginseng medium of the fungus *Daldinia eschscholzii* JC-15, which exhibited acetylcholinesterase inhibitory activity at the concentration of 50 μM with an inhibition rate of 38.8% [41]. One new compound, karimanone (**86**), and two known compounds, (*S*)-5-hydroxy-2-methyl-4-chromanone (**87**) and 5,7-dihydroxy-2-methyl-4-chromanone (**88**), were isolated from the fungus *Daldinia eschscholtzii* KJMT FP4.1 (Karimonjawa National Park). Compounds **87** and **88** were active against a multidrug-resistant strain of *Salmonella enterica* ser., with MIC values of 125, 62.5 and 125 μg/mL, respectively [42]. One new compound, (*R*)-5-hydroxy-8-methoxy-2-methylchroman-4-one (**89**), was isolated from the broad-leaved tree-derived fungus *Daldinia eschscholtzii* (Deyang City, Sichuan, China) [43]. Three new compounds, 7-hydroxy-5-methoxy-2,3-dimethylchromone (**90**), 5-methoxy-2-propanone (**91**), 7-ethyl-8-hydroxy-6-methoxy-2,3-dimethylchromone (**92**), and three known compounds 2,3-dihydro-5-methoxy-2-methylchromen-4-one (**93**), 8-methoxy-2-methyl-4*H*-1-benzopyran-4-one (**94**), 7-hydroxy-2,5-dimethyl-4*H*-1-benzopyran-4-one (**95**), were isolated from mangrove *Bruguiera sexangula* var. *rhynchopetala*-derived fungus *Daldinia eschscholtzii* HJ004 (Hainan, China). Compounds **90** and **93** exhibited *α*-glucosidase inhibitory activity with IC_50_ values of 13.0 and 15.0 μM, respectively [44]. Two new compounds, (5*R*,7*R*)-5,7-dihydroxy-2-methyl-5,6,7,8-tetrahydro-4*H*-chromen-4-one (**96**), (5*R*,7*R*)-5,7-dihydroxy-2-copy-5,6,7,8-tetrahydro-4*H*-chromen-4-one (**97**), along with two known compounds, (5*R*,7*R*)-5,7-dihydroxy-2-methyl-5,6,7,8-tetrahydro-4*H*-chromen-4-one (**98**), (5*R*,7*S*)-5,7-dihydroxy-2-methyl-5,6,7,8-tetrahydro-4*H*-chromen-4-one (**99**), were isolated from lichens-derived fungus *Daldinia* sp. CPCC 400770 (Laojunshan, Dali Bai Autonomous Region, Yunnan, China). Compounds **96** and **98** exhibited significant anti-influenza A virus (IAV) activities, with IC_50_ values of 16.1 and 9.0 μM, respectively [45]. One new compound, 8-ethyl-7-hydroxy-5-carboxyl-2,3-dimethylchromone (**100**), and three known compounds, 5-hydroxy-2-methyl-4*H*-chromen-4-one (**101**), 5,8-dihydroxy-2-methylchromone (**102**) and 5,7-dihydroxy-2-propylchromone (**103**), were isolated from mangrove *B. sexangula* var. *rhynchopetala*-derived fungus *Daldinia eschscholtzii* HJ004 (South China Sea, China). Compounds **100**–**103** showed significant antioxidant activity, with IC_50_ values of 195.03, 20.79, 8.90 and 5.57 μM, respectively, which were stronger than the positive control trolox, with the IC_50_ value of 292.12 μM [46]. One new compound, 3,5-dihydroxy-2-methyl-4*H*-chromen-4-one (**104**), was isolated from the medicinal plant *Pogostemon cablin*-derived fungus *Daldinia eschscholtzii* A630 (Yangchun, Guangdong province, China) [47]. One known compound, (2*R*,4*R*)-3,4-dihydro-5-methoxy-2-methyl-2*H*-1-benzopyran-4-ol (**105**), was isolated from mangrove *Pluchea indica*-derived fungus *Daldinia eschschiltzii* KBJYZ-1 (Zhanjiang, Guangdong Province, China) [48]. One known compound, 5-hydroxy-8-methoxy-2-methyl-4*H*-1-benzopyran-4-one (**106**), was isolated from the mangrove plant *Ceriops tagal*-derived fungus *Daldinia eschscholzii* MCZ-18 (Dong Zhai Gang-Mangrove Garden on Hainan Island, China) [26]. Eight new polyketides, daldinrin A–H (**107**–**114**), were isolated from the *Colletotrichum sinensis*-derived fungus *Daldinia eschscholtzii* cocultured fermentation products with *Colletotrichum pseudomajus* (Xishuangbanna of Yunnan Province, China). Compounds **108** and **109,** with the unique framework of a benzopyran-C_7_ polyketone hybrid, and compounds **110**–**113** were characterized as novel benzopyran dimers. Compound **109** exhibited significant inhibitory activity against the fungi *C*. *pseudomajus* and *Alternaria* sp., with the same MIC value of 1.0 μg/mL, which was stronger than the positive control nystatin. The ether bond at C-4 of the benzopyran derivative increased the antifungal activity [49] (Figure 7).

#### 2.3.3. Lactones

**Figure 8:**
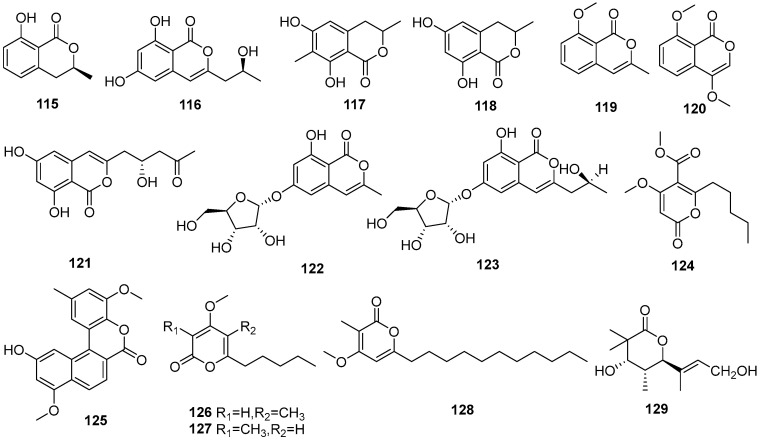

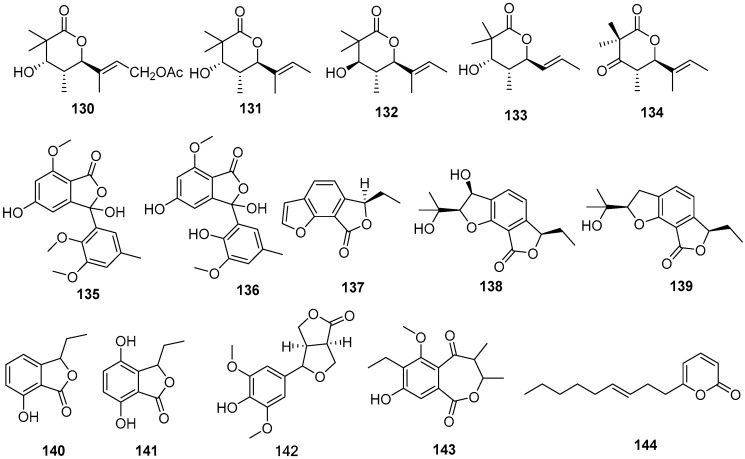
Lactones produced by *Daldinia* species (**115**–**144**).

One known compound, (+)-mellein (**115**), was isolated from the medicinal plant *Cinnamomum bejolghota*-derived fungus *Daldinia eschscholtzii* SDBRCMUNKC745 (Chiang Mai, Thailand). Compound **115** showed *α*-glucosidase inhibitory activity with an IC_50_ value of 0.76 mM [37]. One known compound, (+)-orthosporin (**116**), was isolated from the terrestrial orchid *Paphiopedilum exul*-derived fungus *Daldinia eschscholtzii* (Chiang Mai, Thailand). Compound **116** showed weak activity towards HUVEC cells, with a GI_50_ value of 90.7 μM, and weak cytotoxicity towards HeLa cells, with a CC_50_ value of 89.0 μM [50]. One new pyrone daldineschscin B (**128**), and two known compounds, (3*R*)-methyl-6,8-dihydroxy-7-methyl-3,4-dihydroisocoumarin (**117**) and 6-hydroxymellein (**118**), were isolated from the *Euphorbia pekinensis* endophytic fungus *Daldinia eschscholtzii* J11 (Xishuang Banna Tropical Botanical Garden, Yunnan Province, China) [40]. Three known compounds, 2-acetyl-7-methoxybenzofuran (**119**), 4,8-dimethoxy-1*H*-isochromen-1-one (**120**) and (+)-citreoisocoumarin (**121**), were isolated from Chinese mangrove plant *Ceriops tagal*-derived fungus *Daldinia eschscholzii* MCZ-18 (Dong Zhai Gang-Mangrove Garden on Hainan Island, China). Compound **119** showed broad ranges of antimicrobial spectrum against four pathogenic bacteria, *Enterococcus faecalis*, Methicillin-resistant *Staphylococcus aureus*, *Escherichia coli* and *Pseudomonas aeruginosa*, with MIC values of 12.5, 25, 25 and 12.5 μg/mL, respectively [26]. Two new lactones, daldinisides B (**122**) and C (**123**), were isolated from the fungus *Daldinia eschschilzii* collected from branches of *Scaevola serica* in Hainan [35]. One new daldiniapyrone, 4-methoxy-5-carboxymethoxy-6-pentyl-2*H*-pyran-2-one (**124**), was isolated from the European ash-derived fungus *Daldinia concentrica* (the state of North Rhine Westphalia, Germany) [25]. Three new compounds, childinins A (**125**), F (**126**) and G (**127**), were isolated from the fungus *Daldinia childiae* collected from Kunming Botanical Garden. Compounds **125**–**127** inhibited the growth of *S. aureus*, *S. enterica* sub, *Pseudomonas aeruginosa* at the concentration of 128 μg/mL. Compound **125** showed significant anti-inflammatory activity, with an IC_50_ value of 21.2 μM, and weak activities against SMMC-7721, MCF-7 and SW480 cell lines [33]. Three new lactones, helicascolides D (**129**), E (**130**), and daldinione E (**143**), and two known metabolites, helicascolides A (**131**) and B (**132**), were isolated from mangrove *Bruguiera sexangula* (Lour.) *Poir*-derived fungus *Daldinia eschscholtzii* HJ004 (Hainan, China). Compound **131** had inhibitory activity against *α*-glucosidase, with an IC_50_ value of 16 μM [44]. One new compound, helicascolide G (**133**), was isolated from mangrove *B. sexangula* var. *rhynchopetala*-derived fungus *Daldinia eschscholtzii* HJ004 (South China Sea, China) [46]. One new compound, helicascolide A (**134**), was isolated from the red algae-derived fungus *Daldinia eschscholzii* (South Sulawesi Island, Indonesia). Compound **134** showed fungistatic activity at 200 mg/78.5 mm^2^ spot against plant phytopathogenic fungus *Cladosporium cucumerinum* [51]. Two new phthalides, daldinolides A (**135**) and B (**136**), were isolated from *Daldinia concentrica* (Yamanashi Prefecture, Japan) [30]. One new benzofuran lactone, concentricolide (**137**), was isolated from the fungus *Daldinia concentrica* collected from Lijiang of Yunnan. Compound **137** inhibited HIV-1-induced cytopathic effects, with an EC_50_ value of 0.31 μg/mL, and exhibited the blockage with the EC_50_ value of 0.83 µg/mL, on syncytium formation between HIV-1 infected cells and normal cells [52]. Two new compounds, daldinins A (**138**) and B (**139**), along with two known compounds isoochracein (**140**) and 4-hydroxyisoochracein (**141**), were isolated from fungus *Daldinia concentrica* (Laojunshan, Yunnan Province, China) [53]. One known compound, curuilignan D (**142**), was isolated from the European ash-derived fungus *Daldinia concentrica* (the state of North Rhine Westphalia, Germany) [25]. One new compound, (*E*)-6-(non-3-en-1-yl)-2*H*-pyran-2-one (**144**), was isolated from broad-leaved tree-derived fungus *Daldinia eschscholtzii* (Deyang City, Sichuan Province, China) [43] (Figure 8).

#### 2.3.4. Naphthalenone Derivatives

**Figure 9:**
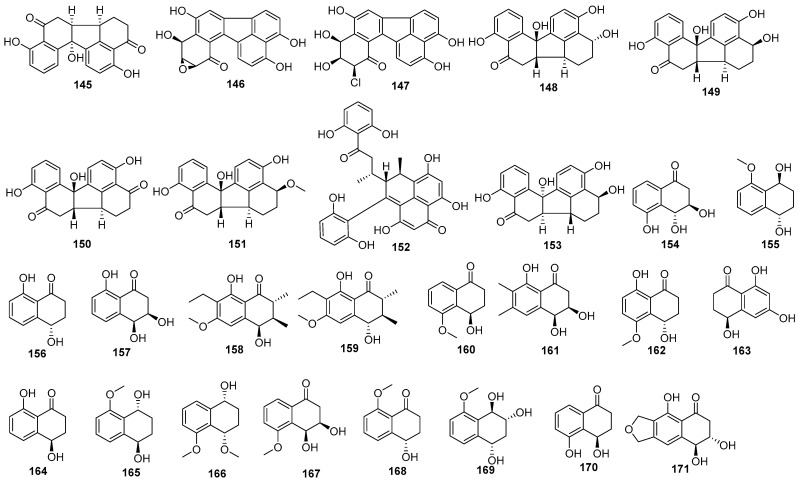
Naphthalenone derivatives produced by *Daldinia* species (**145**–**171**).

One new compound, daldinone A (**145**), was isolated from the European ash-derived fungus *Daldinia concentrica* (North Rhine Westphalia, Germany) [25]. A new biosynthetically related epoxide-containing daldinone analog, daldinone B (**146**), and a new chlorinated pentacyclic polyketide daldinone E (**147**), were purified from the *Chrysophyllum cainito*-derived fungus *Daldinia* sp. treated with the epigenetic modifier suberoylanilide hydroxamic acid (SAHA). Compounds **146** and **147** exhibited DPPH radical scavenging activities, with IC_50_ values of 3.1 and 3.6 μM, respectively, comparable to the positive control ascorbic (IC_50_ = 3.2 μM) [54]. Two new compounds, daldinones F (**148**) and G (**149**), and two known compounds, daldinones C (**150**) and D (**151**), were isolated from the orchid *Paphiopedilum exul* (Ridl.) Rolfe-derived fungus *Daldinia eschscholtzii* (Chiang Mai, Thailand). Compounds **150** and **151** showed cytotoxic activity towards SW1116 cells, with IC_50_ values of 41.0 and 49.5 μM, respectively, compared with 37.0 μM for 5-fluorouraci [50]. Two new polyketides, dalmanol C (**152**) and daldinone F (**153**), were isolated from *Tenodera aridifolia*-derived fungus *Daldinia eschscholzii* (Purple Mountain, Nanjing, China). Compound **152** exhibited moderate cytotoxic activity against human colon adenocarcinoma cell line SW480, with an IC_50_ value of 9.59 μM [38]. One known compound, 3,4,5-trihydroxy-1-tetralone (**154**), was isolated from the European ash-derived fungus *Daldinia concentrica* (the state of North Rhine Westphalia, Germany) [25]. One new polyketide derivative, (1*R*,4*R*)-5-methoxy-1,2,3,4-tetrahydronaphthalene-1,4-dio (**155**), was isolated from mangrove *Pluchea indica* Less-derived fungus *Daldinia eschschiltzii* KBJYZ-1 (Zhanjiang, Guangdong Province) [48]. Two known compounds, (+)-regiolone (isosclerone) (**156**) and (+)-3,4-dihydro-3,4,8-trihydroxy-1(2*H*)-naphthalenone (**157**), were isolated from *Cinnamomum bejolghota*-derived fungus *Daldinia eschscholtzii* SDBRCMUNKC745 (Chiang Mai, Thailand). Compounds **156** and **157** showed *α*-glucosidase inhibitory activity, with IC_50_ values of 0.85 and 0.55 mM, respectively [37]. Two new compounds, daldiniones C–D (**158**–**159**), and two known compounds, 4*R*-(O)-methylsclerone (**160**) and (−)-cis-(3R*,4S*)-3,4,8-trihydroxy-6,7-dimethyl-3,4-dihydronaphthalen-1(2*H*)-one (**161**), were isolated from *B. sexangula* var. *rhynchopetala*-derived fungus *Daldinia eschscholtzii* HJ004 (the South China Sea, China). Compound **161** exhibited *α*-glucosidase inhibitory activities with IC_50_ values of 21 μM [44]. One new polyketone, (4R*)-4,8-dihydroxy-3-hydro-5-methoxy-1-naphthhthalenone (**162**), was isolated from the oak leaves-derived fungus *Daldinia* sp. (Tokushima) [21]. One known compound, 4,6,8-trihydroxide-3,4-dihydronaphthhalen-1(2*H*)-one (**163**), was isolated from the branches of *Scaevola sericea* Vahl-derived fungus *Daldinia eschscholzii* (Hainan, China) [35]. One known compound, (-)-regiolone (**164**), was isolated from the lichen *Punctelia* sp.-derived fungus *Daldinia childiae* (Cloak Mountain Scenic Area, Guizhou Province, China) [15]. One new polyketide derivative, (1*R*,4*R*)-5-methoxy-1,2,3,4-tetrahydronaphthalene-1,4-dio (**165**), was isolated from the mangrove *Pluchea indica* Less-derived fungus *Daldinia eschschiltzii* KBJYZ-1 (Zhanjiang, Guangdong Province) [48]. One new polyketide, daldinrin K (**166**), was isolated from the antagonistic coculture with tea phytopathogen *Colletotrichum pseudomajus,* which induces antifungal cryptic metabolites from isogenesis endophyte *Daldinia eschscholtzii* against tea phytopathogens. Compound **166** showed anti-feedant activities against silkworms with a feeding deterrence index of 81%. Positive control, abamectin, had antifeedant activity against the silkworms with a feeding deterrence index of 100% at concentrations of 50 μg/cm^2^ [49]. One new naphthalene derivative, dalesconoside F (**167**), and four known compounds, nodulisporone (**168**), xylariol A (**169**), (4*R*)-4,8-dihydroxy-3-hydro-5-methoxy-1-naphthalenone (**162**), (4*R*)-3,4-dihydro-4,5-dihydroxynaphthalen-1(2*H*)-one (**170**), were isolated from mangrove *Ceriops tagal*-derived fungus *Daldinia eschscholzii* MCZ-18 (Dong Zhai Gang-Mangrove Garden on Hainan Island, China). Compounds **162** and **170** showed broad ranges of antimicrobial spectrum against five indicator test microorganisms, *E. faecalis*, Methicillin-resistant *S. aureus*, *E. coli*, *P. aeruginosa* and *C. albicans*. Compound **162** exhibited antimicrobial activities with MIC values of 25, 12.5, 12.5, 25 and 25 μg/mL, respectively. Compound **170** exhibited antimicrobial activities with MIC values of 50, 25, 50, 25 and 25 μg/mL, respectively [26]. One known compound, nodulone (**171**), was isolated from the orchid-associated derived fungus *Daldinia eschscholtzii* (Family Xylariaceae) from the terrestrial orchid *Paphiopedilum exul* (Ridl.) Rolfe (collected Chiang Mai Thailand) [50] (Figure 9).

#### 2.3.5. Other Polyketides

**Figure 10:**
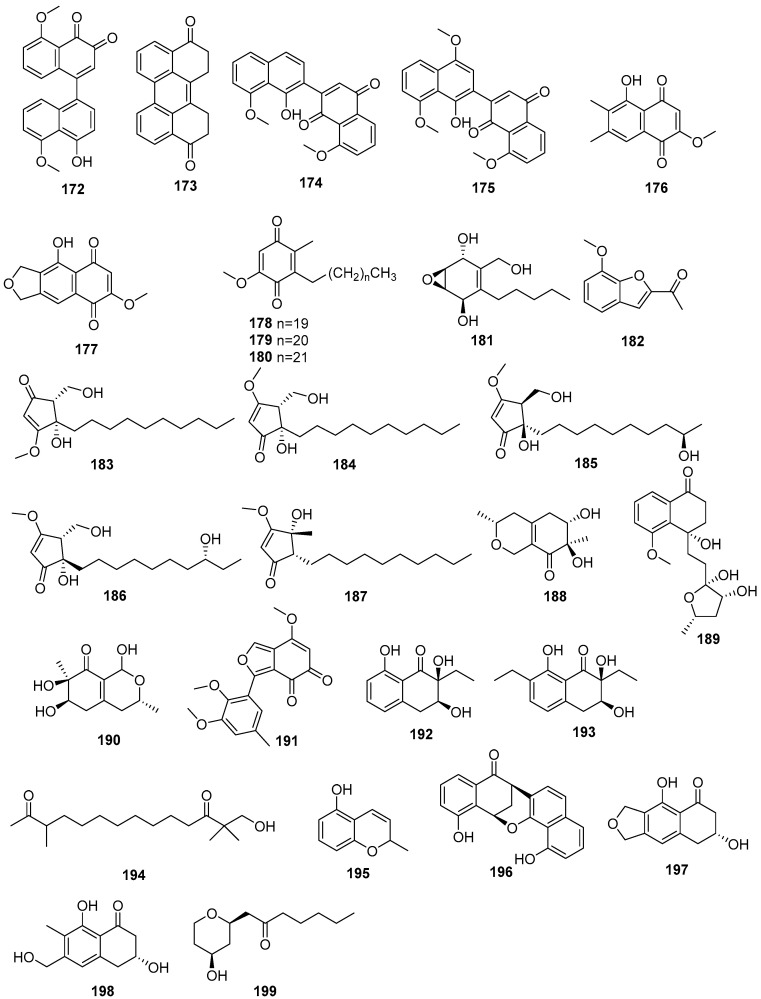
Other polyketides produced by *Daldinia* species (**172**–**199**).

**Scheme 7:**
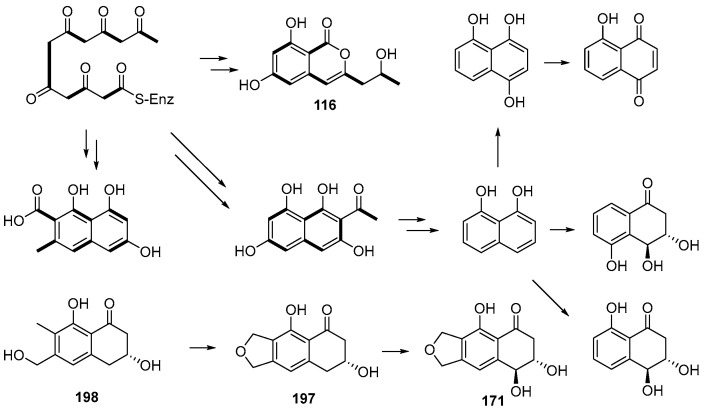
Putative biosynthetic pathway of polyketides (**116**, **171**, **197**–**198**) [50].

One new naphthoquinone, daldiquinone (**172**), was isolated from *Daldinia concentrica* collected in Yamanashi Prefecture, Japan. Compound **172** exhibited anti-angiogenesis activity against human umbilical vein endothelial cells, with an IC_50_ value of 7.5 μM [30]. One new polyketide derivative, dalditene A (**173**), was isolated from the mangrove *Pluchea indica* Less-derived fungus *Daldinia eschschiltzii* KBJYZ-1 (Guangdong Province, China). Compound **173** exhibited significant anti-inflammatory activity, inhibiting the expression of iNOS and COX-2 in LPS-induced RAW264.7 cells with an IC_50_ value of 12.9 μM [48]. One new naphthalene derivative dalesconoside D (**174**), and a known synthetic analog dalesconoside E (**175**), were isolated from Chinese mangrove plant *C. tagal*-derived fungus *Daldinia eschscholzii* MCZ-18 (Dong Zhai Gang-Mangrove Garden on Hainan Island, China) [26]. Two new compounds, 5-hydroxy-2-methoxy-6,7-dimethyl-1,4-naphthoquinone (**176**) and 5-hydroxy-2-methoxynaphtho[9–*c*]furan-1,4-dione (**177**), were obtained from the mangrove *B. sexangula* var. *Rhynchopetala*-derived fungus *Daldinia eschscholtzii* HJ004 (South China Sea, China). Compound **177** displayed a potent inhibitory activity against *α*-glucosidase with an IC_50_ value of 5.7 µg/mL. Compound **176** exhibited antibacterial activity against *B. cereus* with an MIC value of 12.5 µg/mL [39]. Three new compounds, 3-alkyl-5-methoxy-2-methyl-1,4-benzoquinones,3-heneicosyl (**178**), 3-docosyl-5-methoxy-2-methyl-1,4-benzoquinone (**179**) and 5-methoxy-2-methyl-3-tricosyl-1,4-benzoquinone (**180**), were isolated from the fruiting bodies of *Daldinia concentrica* (Laojunshan, Yunnan, China) [55]. One new polyketide, daldinrin J (**181**), was isolated from the antagonistic coculture with tea phytopathogen *C. pseudomajus*-induced antifungal cryptic metabolites from isogenesis endophyte *D. eschscholtzii* against tea phytopathogens. Compound **181** exhibited anti-feeding activity against silkworms with a feeding deterrence index (FDI) of 81% at the concentration of 50 μg/cm^2^ [49]. One known compound, 2-acetyl-7-methoxybenzofuran (**182**), was obtained from the mangrove *B. sexangula* var. *rhynchopetala*-derived fungus *Daldinia eschscholtzii* HJ004 from the South China Sea. Compound **182** displayed potent inhibitory activity against *α*-glucosidase with the IC_50_ value of 1.1 µg/mL [39]. Two new compounds, daldisones A (**183**) and B (**184**), were isolated from the fungus *Daldinia* sp. CPCC 400770. Compounds **183** and **184** exhibited significant anti-influenza virus activity, with IC_50_ values of 16.0 and 7.4 μM, respectively. Compound **184** showed moderate antibacterial activities against *S. aureus*, *Enterococcus faecalis* and *B. cereus* with MIC values of 32.0, 16.0 and 32.0 μg/mL, respectively [56]. To activate or overproduce secondary metabolites, the native promoter of *LaeA-like* in *Daldinia eschscholzii* was substituted by a strong gpdA promoter (amplified from the *Aspergillus nidulans* genomic DNA), led to the generation of two novel cyclopentenone metabolites, dalestones A (185) and B (**186**). Compounds **185** and **186** inhibited the expression of TNF-*α* and IL-6 in LPS-induced RAW264.7 macrophages [57]. One new cyclopentenone, daldineschscin A (**187**), together with one known compound, xylariphilone (**188**), were isolated from *Euphorbia pekinensis* endophytic fungus *Daldinia eschscholtzii* J11. Compound **188** possessed moderate ABTS+ radical scavenging activity with an IC_50_ value of 49.13 ± 0.62 μM, compared with the positive control vitamin C, with an IC_50_ value of 18.27 ± 1.04 μM [40]. One new naphthalene derivative, dalesconoside B (**189**), and one known compound, fusaraisochromenone (**190**), were isolated from Chinese mangrove plant *C. tagal*-derived fungus *Daldinia eschscholzii* MCZ-18 (Dong Zhai Gang-Mangrove Garden on Hainan Island, China), and compound **189** bore a rare ribofuranoside substituted at C-1 and the 5-methyltetrahydrofuran-2,3-diol moiety. Compound **190** showed antibacterial activity against *E. faecalis*, Methicillin-resistant *S. aureus*, *E. coli*, *P. aeruginosa* and *C. albicans,* with MIC values of 12.5, 12.5, 6.25, 12.5 and 25 μg/mL, respectively [26]. One new compound, childinin B (**191**), was isolated from the fungus *Daldinia childiae* from Kunming Botanical Garden. Compound **191** showed exhibited remarkable activity against *S. aureus* with an MIC_90_ value of 54.9 μg/mL [33]. Two new compounds, daldiniones A–B (**192**–**193**), were isolated from the fungus *Daldinia eschscholtzii* HJ004 from *B. sexangula* var. *Rhynchopetala* in Hainan [44]. Two new compounds, eschscholin A (**194**) and 3-ene-2-methyl-2*H*-1-benzopyran-5-ol (**195**), were isolated from the medicinal plant *Pogostemon cablin* endophytic fungus *Daldinia eschscholtzii* A630 [47]. Three new compounds, daldionin (**196**), nodulones B (**197**) and C (**198**), were isolated from the orchid-associated fungal strain *Daldinia eschscholtzii*. The structures of the polyketide metabolites suggest joint biogenetic origins and expand on the known possible reaction channels from the 1,8-dihydroxynaphthalene (DHN) pathway of melanin biosynthesis (Scheme 7). Compound **196** had weak anti-proliferative activity against HUVEC (GI_50_ 98.4 μM) and K-562 cell lines (GI_50_ 85.5 μM) [50]. One known compound, pyranlyketide (**199**), was isolated from the antagonistic co-culture with tea phytopathogen *Colletotrichum pseudomajus* induced antifungal cryptic metabolites from isogenesis endophyte *Daldinia eschscholtzii* [49] (Figure 10).

### 2.4. Polyphenols

#### 2.4.1. Naphthols

**Figure 11:**
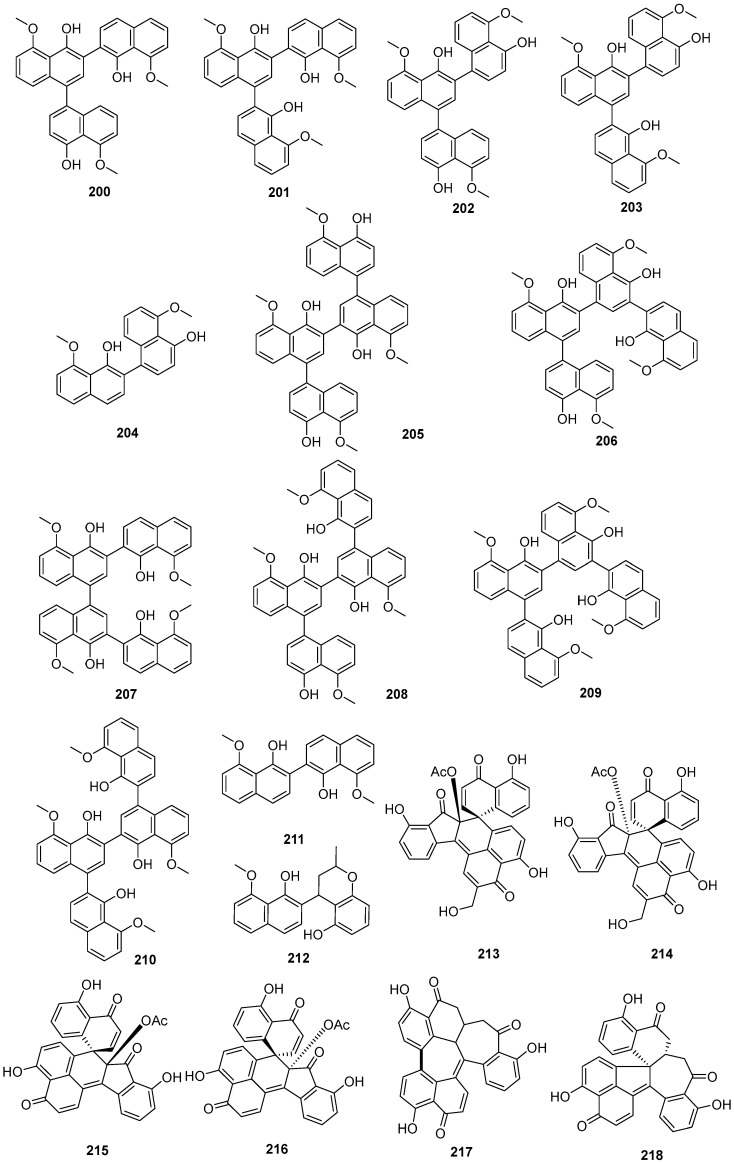

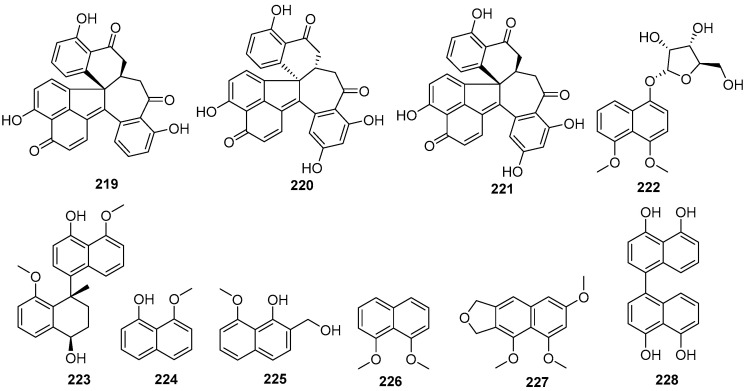
Naphthols produced by *Daldinia* species (**200**–**228**).

**Scheme 8:**
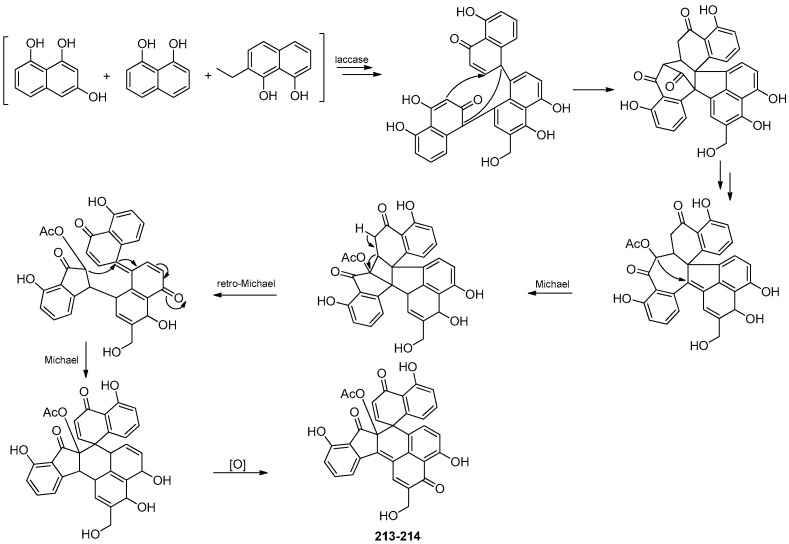
Putative biosynthetic pathway of (±) galewone (**213**–**214**) [60].

Four new compounds, 1,1′,3′,3″-ternaphthalene-5,5′,5″-trimethoxy-4,4′,4″-triol (**200**), 3,1′,3′,3″-ternaphthalene-5,5′,5″-trimethoxy-4,4′,4″-triol (**201**), 1,1′,3′,1″-Ternaphthalene-5,5′,5″-trimethoxy-4,4′,4″-triol (**202**), 3,1′,3′,1″-Ternaphthalene-5,5′,5″-trimethoxy-4,4′,4″-triol (**203**), and one known compound nodulisporin A (**204**), were isolated from the lichen *Parmelia adaugescens*-derived fungus *Daldinia childiae* 047215 (Mt. Halla, Jeju Island, South Korea). Compounds **201** and **204** exhibited concentration-dependent adiponectin synthesis-promoting activity, with EC_50_ values of 30.8 and 15.2 μM, respectively, and had bounded to three peroxisome proliferator-activated receptor (PPAR) subtypes (PPAR*α*, PPAR*γ*, and PPAR*δ*). In addition, compound **201** transactivated retinoid X receptor *α*, whereas **204** did not. Naphthol oligomers **201** and **204** represented novel pan-PPAR modulators and were potential pharmacophores for designing new therapeutic agents against hypoadiponectinemia-associated metabolic diseases [58]. Six new compounds 1,1′,3′,3″,1″,1‴-quaternaphthalene-5,5′,5″,5‴-tetramethoxy-4,4′,4″4‴-tetraol (**205**), 1,1′,3′,1″,3″,3‴-quaternaphthalene-5,5′,5″,5‴-tetramethoxy-4,4′,4″4‴-tetraol (**206**), 3,3′,1′,1″,3″,3‴-quaternaphthalene-5,5′,5″,5‴-tetramethoxy-4,4′,4″4‴-tetraol (**207**), 1,1′,3′,3″,1″,3‴-quaternaphthalene-5,5′,5″,5‴-tetramethoxy-4,4′,4″4‴-tetraol (**208**), 3,1′,3′,1″,3″,3‴-quaternaphthalene-5,5′,5″,5‴-tetramethoxy-4,4′,4″4‴-tetraol (**209**), 3,1′,3′,3″,1″,3‴-quaternaphthalene-5,5′,5″,5‴-tetramethoxy-4,4′,4″4‴-tetraol (**210**), and two known compounds nodulisporins B (**211**) and E (**212**), were isolated from lichen *Parmelia adaugescens*-derived fungus *Daldinia childiae* 047219 (Hannah Mountain in Jeju Island, South Korea). Feature-based molecular networking analysis suggested six new tetramers (**205**–**210**) were composed of the 5-methoxy-4-naphthol skeleton, each of which was connected to one another in various positions. Compound **212** had significant NO inhibitory effects on LPS-induced RAW cells, reducing NO production by more than 50% at the concentration of 25 and 50 μM, respectively [59]. A pair of novel polyphenolic compounds with undescribed seven-ring frameworks of the carbon skeleton, (-)-galewone (**213**) and (+)-galewone (**214**), were isolated from mantis *Tenodera aridifolia*-derived fungus *Daldinia eschscholzii* IFB-TL01 and proposed a possible pathway for its formation (Scheme 8). Compounds **213** and **214** were observed to reduce the cell viability of activated hepatic stellate cells CFSC-8B with IC_50_ values of 3.73 ± 0.21 and 10.10 ± 0.41 μM, respectively [60]. A pair of new compounds, (+)-spirodalesol (**215**) and (-)-spirodalesol (**216**), were isolated from *Daldinia eschscholzii* residing in the mantis *Tenodera aridifolia* gut. Compounds **215** and **216** significantly suppressed the secretion of IL-1*β* with IC_50_ values of 0.67 and 0.70 μM, respectively, which indicated approximately 30-fold greater activity than the positive control andrographolide (IC_50_ = 21.53 μM) [61]. One new compound, selesconol (**217**), was isolated from *Daldinia eschscholzii* IFB-TL01 as an inducer for the differentiation of rat bone marrow mesenchymal stem cells into neural cells [62]. Two pairs of new enantiomers, (±)-dalesconols A and B, with an unprecedented carbon skeleton, were isolated from the gut of the mantis species *Tenodera aridifolia*-derived fungus *Daldinia eschscholzii* IFB-TL01. Optical resolution of (±)-dalesconol A and (±)-dalesconol B by HPLC on a chiral phase gave the corresponding enantiomers, (+)-dalesconol A (**218**), (-)-dalesconol A (**219**) and (+)-dalesconol B (**220**), (-)-dalesconol B (**221**) [63]. Two new naphthalene derivatives, dalesconosides A (**222**) and C (**223**), were isolated from mangrove plant *C. tagal*-derived fungus *Daldinia eschscholzii* MCZ-18 (Dong Zhai Gang-Mangrove Garden on Hainan Island, China). Compound **222** was bearing a rare ribofuranoside substituted at C-1 and the 5-methyltetrahydrofuran-2,3-diol moiety. Compound **222** showed broad ranges of the antimicrobial spectrum against five pathogenic bacteria, *E. faecalis*, Methicillin-resistant *S. aureus*, *E. coli*, *P. aeruginosa* and *C. albicans*, with MIC values of 6.25, 25, 12.5, 6.25 and 25 μg/mL, respectively. Compound **223** showed antimicrobial activity against *E. faecalis*, Methicillin-resistant *S. aureus*, *E. coli* and *P. aeruginosa* with MIC values of 25, 25, 25 and 12.5 μg/mL, respectively [26]. One known compound, 8-methoxynaphthalen-1-ol (**224**), was isolated from the fungus *Daldinia loculata* collected from Laojunshan in Yunnan Province [64]. One new compound, daldiniol G (**225**), was isolated from the medicinal plant *Anoectochilus roxburghi* endophytic fungus *Daldinia* sp. TJ403-LS1 (Wuhan, Hubei Province, China). Compound **225** showed good butyrylcholinesterase (BChE) inhibitory activity, with an IC_50_ value of 15.53 ± 0.39 μM, better than the positive control neostigmine with an IC_50_ value of 49.60 ± 6.10 μM. Molecular docking was performed to investigate the binding mode of compound **225** to BChE. Three-dimensional models showed that the active compound **225** fitted well in the active pocket of BChE surrounded with Asp70, Trp82, Gly115, Gly116, Glu197, Tyr332, Tyr472 and His438. Further analysis revealed that compound **225** formed hydrogen bonds with Glu197, and their benzene ring structure formed π-π conjugation with Trp82 and cation-π interaction with potassium ion [65]. One known compound, 1,8-dimethoxynaphthalene (**226**), was isolated from the *Euphorbia pekinensis* endophytic fungus *Daldinia eschscholtzii* J11 (Xishuang Banna Tropical Botanical Garden, Yunnan Province, China) [40]. One new naphthofuran, 1,3,8-trimethoxynaphtho[9–*c*]furan (**227**), was obtained from mangrove *B. sexangula* var. *Rhynchopetala*-derived fungus *Daldinia eschscholtzii* HJ004 (South China Sea, China) [39]. One known compound, 4:5:4′:5′-tetrahydroxy-1:1′-binaphthyl (**228**), was isolated from the European ash-derived fungus *Daldinia concentrica* (the state of North Rhine Westphalia, Germany) [25] (Figure 11).

#### 2.4.2. Phenolics

**Figure 12:**
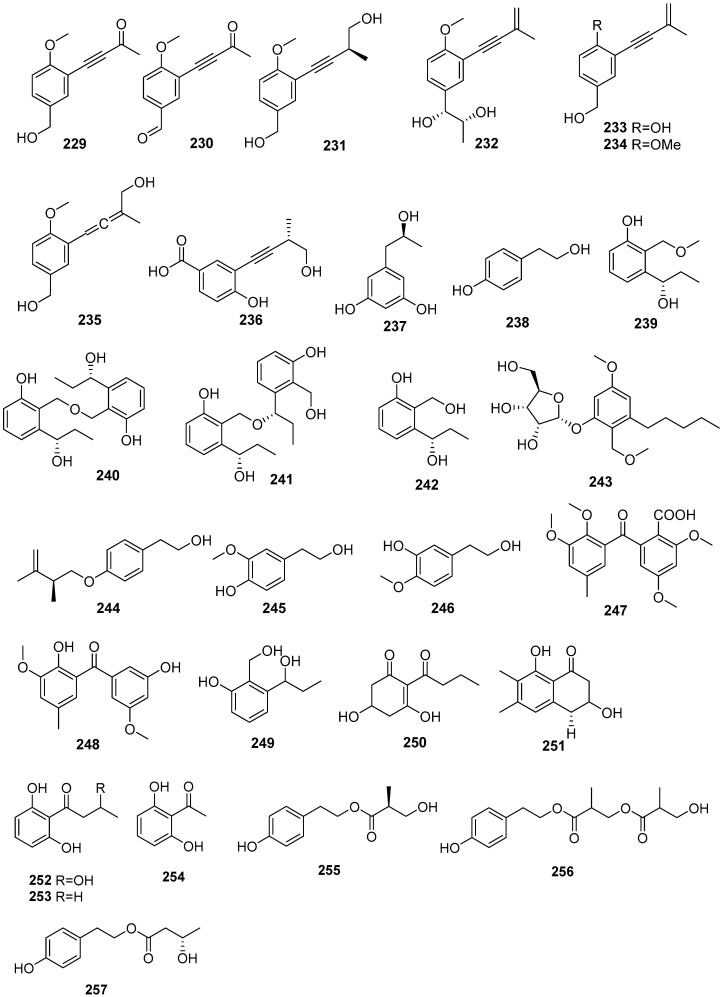
Phenolics produced by *Daldinia* species (**229**–**257**).

Five new phenolics, daldiniols A–E (**229**–**232**, **235**), and two known analogs, 4-hydroxy-3-(3-methylbut-3-en-l-ynyl) benzyl alcohol (**233**) and 4-methoxy-3-(3-methylcut-3-en-l-ynyl) benzyl alcohol (**234**), were isolated from medicinal plant *Nelumbo nucifera* endophytic fungus *Daldinia* sp. TJ403-LS1. Compound **229** exhibited remarkable immunosuppressive activity against LPS and anti-CD3/anti-CD28 mAbs-activated murine splenocyte proliferation with the same IC_50_ value of 0.06 μM and BChE inhibitory activity with an IC_50_ value of 6.93 ± 0.71 μM. Compound **234** showed potent BChE inhibitory activity, with an IC_50_ value of 16.00 ± 0.30 μM, better than the positive control neostigmine (IC_50_ = 49.60 ± 6.10 μM). Molecular docking was performed to investigate the binding mode of compounds **229** and **234** to BChE. Three-dimensional models showed that the active compounds **229** and **234** fitted well in the active pocket of BChE surrounded with Asp70, Trp82, Gly115, Gly116, Glu197, Tyr332, Tyr472 and His438. Further analysis revealed that compound **225** formed a hydrogen bond with Glu197, and their benzene ring structure formed π-π conjugation with Trp82 and cation-π interaction with potassium ion. These two compounds could also form π-π conjugate with His438 and form additional hydrogen bonds with Tyr128 and Ser198, respectively [65]. One new polyketide derivative, dalditone B (**236**), was isolated from the mangrove-derived fungus *Daldinia eschschiltzii* KBJYZ-1 (Zhanjiang, Guangdong Province, China) [48]. One known compound, orcinotriol (**237**), was isolated from *Scaevola serica*-derived fungus *Daldinia eschscholzii* (Hainan, China) [35]. One known compound, 4-(2-hydroxyethyl) phenol (**238**), was isolated from the fungus *Daldinia loculata*, which was collected from Laojunshan in Yunnan Province, China [64]. Three new benzenes daldins, A–C (**239**–**241**), along with one known benzene derivative, 2-hydroxymethyl-3-(1-hydroxypropyl) phenol (**242**), were isolated from fruiting bodies of fungus *Daldinia concentrateca* S0318 (Laojunshan, Yunnan Province, China) [66]. One new polyketide, daldinrin L (**243**), was isolated from the co-cultured fermentation products from *Colletotrichum pseudomajus* and *Daldinia eschscholtzii*. Compound **243** exhibited anti-feeding activity against silkworms with a feeding deterrence index (FDI) of 70% at the concentration of 50 μg/cm^2^, while the positive control, abamectin, had anti-feedant activity against the silkworms with the FDI of 100% at the concentration of 50 μg/cm^2^ [49]. Three known phenolic compounds, stachyline C (**244**), 3-methoxy-4-hydroxyphenylethanol (**245**) and 3-hydroxy-4-methoxy-phenylethanol (**246**), were isolated from lichen-derived fungus *Daldinia* sp. CPCC 400770 (Laojunshan, Dali Bai Autonomous Region, Yunnan Province, China) [45]. Two new compounds, childinins D (**247**) and E (**248**), were isolated from the fungus *Daldinia childiae* from Kunming Botanical Garden [33]. One known compound, 2-(hydroxymethyl)-3-(1-hydroxypropyl) phenol (**249**), was isolated from the fungus *Daldinia concentrica* obtained from Laojunshan in Yunnan Province [53]. One known compound, AB5046A (**250**), was isolated from the fungus *Daldinia eschscholtzii* KJMT FP4.1 (Karimonjawa National Park). Compound **250** was active against the multidrug-resistant strain of *Salmonella enterica* ser. Typhi with an MIC of 125 μg/mL [42]. One new hydronaphthalenone, (3*S*)-3,8-dihydroxy-6,7-dimethyl-*α*-tetralone (**251**), was isolated from the mangrove plant *Bruguiera gymnorrhiza* (L.) Soavigny endophytic fungus *Daldinia eschscholtzii* PSU-STD57 (Suratthani province, Thailand) [67]. Three known compounds, 3-hydroxy-1-(2,6-dihydroxyphenyl)butan-1-one (**252**), 1-(2,6-dihydroxy-phenyl)butan-1-one (**253**) and 1-(2,6-dihydroxyphenyl) ethan-1-one (**254**), were isolated from the *Euphorbia pekinensis* endophytic fungus *Daldinia eschscholtzii* J11. Compounds **252** and **253** possessed moderate ABTS+ radical scavenging activity with IC_50_ values of 43.66 ± 1.36 and 28.08 ± 1.03 μM, respectively, compared with the positive control vitamin C, with the IC_50_ value of 18.27 ± 1.04 μM. Compound **252** showed weak DPPH radical scavenging activity with an IC_50_ value of 182.27 ± 2.42 μM, compared with vitamin C (IC_50_ = 12.29 ± 0.43 μM) [40]. Three new compounds, daldispols A–C (**255**–**257**), were isolated from the lichen-derived fungus *Daldinia* sp. (Laojunshan, Dali Bai Autonomous Region, Yunnan Province, China). Compounds **255** and **257** exhibited significant anti-influenza A virus (IAV) activities, with IC_50_ values of 12.7 and 6.4 μM, respectively [45] (Figure 12).

### 2.5. Steroids

**Figure 13:**
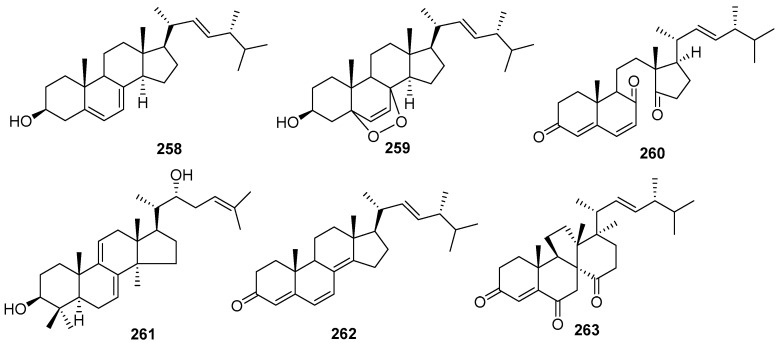
Steroids produced by *Daldinia* species (**258**–**263**).

Steroids are a kind of natural active substance with a wide range of sources. Six steroids were isolated from the genus of *Daldinia* (Figure 13), including two new compounds, and four compounds exhibited cytotoxicity and anti-inflammatory activity.

Two known triterpenoid compounds, ergosterol (**258**) and ergosterol peroxide (**259**), were isolated from the fungus *Daldinia concentrica* collected from a national park in Yee On Province, Vietnam. Compound **258** showed weak cytotoxicity against HepG2, Hep3B and MCF7 cells, with IC_50_ values of 21.5 ± 5.1, 21.7 ± 2.8 and 43.6 ± 5.1 µM, respectively. Compound **259** showed weak cytotoxicity against HepG2 and Hep3B cells, with IC_50_ values 46.9 ± 3.7 and 35.2 ± 3.4 µM, respectively [22]. One new terpenoid, childinasterone A (**260**), was isolated from the fungus *Daldinia Childiae* collected from Kunming Botanical Garden, China. Compound **260** showed significant suppression of NO production in LPS-stimulated RAW264.7 cell activity with IC_50_ value of 21.2 µM, and weak activity against SMMC-7721 (inhibition rate 53.3%), MCF-7 (inhibition rate 64.6%) and SW480 (inhibition rate 67.0%), at the concentration of 40 µM [33]. One new compound, 22*R*-hydroxylanosta-7,9(11),24-trien-3-one (**261**), was isolated from the European ash-derived fungus *Daldinia concentrica* (the state of North Rhine Westphalia, Germany) [25]. One known compound, 22*E*-ergosta-4,6,8(14),22-tetraen-3-one (**262**), was isolated from the fermentation of the *Euphorbia pekinensis* endophytic fungus *Daldinia eschscholtzii* J11 [40]. One known compound, dankasterone A (**263**), was isolated from mangrove plant *C. tagal*-derived *Daldinia eschscholzii* MCZ-18 (Dong Zhai Gang-Mangrove Garden on Hainan Island, China) [26] (Figure 13).

### 2.6. Other Classes

**Figure 14:**
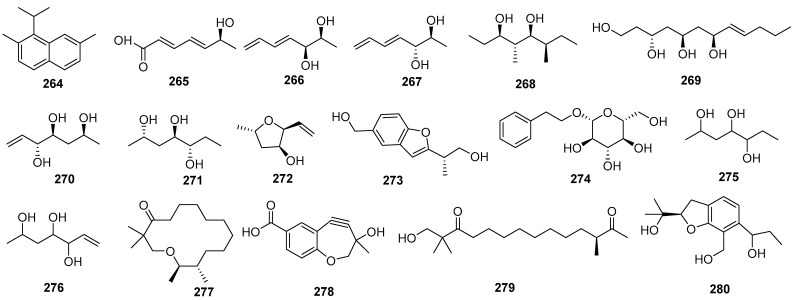
Other classes produced by *Daldinia* species (**264**–**280**).

Seventeen other class compounds were isolated from the fungi *Daldinia* spp., including eleven new compounds, of which eight compounds exhibited cytotoxicity, insect resistance activity and anti-inflammatory activities (Figure 14).

One new compound, 1-isopropy-2,7-dimethylnaphthhalene (**264**), was isolated from the fungus *Daldinia* sp. [24]. Five new compounds, coldols A–C (**265**–**267**), coldiol (**268**) and daldinrin I (**269**), and three known compounds (2S,4S,5R)-hept-6-en-2,4,5-triol (**270**), hypoxylonol H (**271**), 5-methyl-2-vinyltetrahydrofuran-3-ol (**272**), were isolated from the co-cultured fermentation products from *C. pseudomajus* and *Daldinia eschscholtzii*. Compound **269** exhibited anti-feeding activity against silkworms with an FDI of 97% at the concentration of 50 μg/cm^2^. Compounds **268** and **270**–**272** exhibited weak anti-feeding activity against silkworms with FDIs of 20%, 30%, 50% and 15%, respectively, at the concentration of 50 μg/cm^2^ [49]. One new compound, daldiniol F (**273**), was isolated from the medicinal plant *Nelumbo nucifera* endophytic fungus *Daldinia* sp. TJ403-LS1. Compound **273** showed potent BChE inhibitory activity (IC_50_, 23.33 ± 0.55 μM), better than the positive control neostigmine (IC_50_, 49.60 ± 6.10 μM). Molecular docking was performed to investigate the binding mode of compound **273** to BChE. Compound **273** formed hydrogen bonds with Glu197, and their benzene ring structure formed π-π conjugation with Trp82 and cation-π interactions with potassium ions [65]. Compound **273** was also isolated from mangrove *B. sexangula* var. *Rhynchopetala*-derived fungus *Daldinia eschscholtzii* HJ004 (the South China Sea, China) [46]. Compound **273** showed good BChE inhibitory activity, with an IC_50_ value of 23.33 ± 0.55 μM, better than the positive control neostigmine, with an IC_50_ value of 49.60 ± 6.10 μM [66]. One known phenolic compound, 2-phenylethyl-*β*-*D*-glucopyranoside (**274**), was isolated from the lichen-derived fungus *Daldinia* sp. CPCC 400770 (Laojunshan, Dali Bai autonomous prefecture, Yunnan province, China). Compound **274** exhibited significant anti-influenza A virus (IAV) activity with an IC_50_ value of 12.5 μM [45]. One new alkanol, 2,4,5-heptanetriol (**275**), together with one known compound, 6-heptene-2,4,5-triol (**276**), were isolated from the lichen *Punctelia* sp.-derived fungus *Daldinia childiae* (Cloak Mountain Scenic Area in Guizhou Province of China) [15]. Two new polyketide derivatives, eschschilin B (**277**) and dalditone A (**278**), and one known compound, eschscholin A (**279**), were isolated from mangrove *Pluchea indica*-derived fungus *Daldinia eschschiltzii* KBJYZ-1 (Zhanjiang, Guangdong Province, China). Compound **277** exhibited significant anti-inflammatory activity by inhibiting the expression of pP65 and p-I*κ*B*α* proteins in LPS-induced RAW264.7 cells. The anti-inflammatory effect of compound **277** may be connected to the suppressed NF–*κ*B signaling pathways in RAW264.7 cells with an IC_50_ value of 19.3 μM [48]. One new compound, daldinin C (**280**), was isolated from the fungus *Daldinia concentrica* (Laojunshan in Yunnan Province) [53] (Figure 14).

## 3. Conclusions

**Figure 15:**
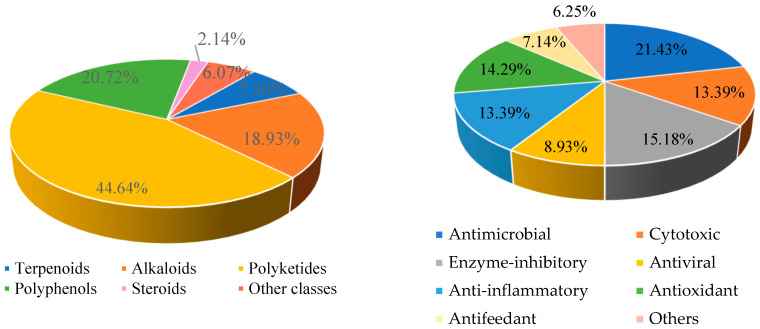
Structural diversity (**left**) and bioactivities (**right**) of compounds isolated from *Daldinia* sp. that were discovered from January 1995 to June 2024.

**Figure 16:**
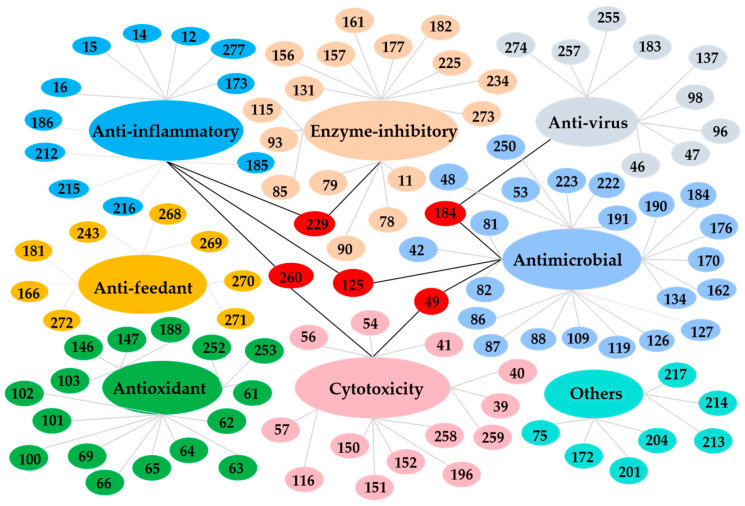
Biological activities of secondary metabolites isolated from *Daldinia* sp.

In this review, the sources, structural diversity and biological activity of secondary metabolites from the fungi *Daldinia* species are summarized, covering the period between January 1995 and June 2024. A total of 280 metabolites, including 196 new compounds, were obtained from the genus *Daldinia*. Remarkably, among them, 18 compounds (**46**–**55**, **74**, **85**, **213**–**214**, **219**–**222**) had novel skeleton structures. Furthermore, 280 compounds, along with their biological activities, producing strains and habitats, are summarized in Table 1. Among the total 47 taxa in the genus *Daldinia*, the fungi *D. eschscholzii* and *D. concentrica* are the two species that have been most extensively studied in chemical investigation. The vast metabolic diversity that may be encountered in a single species or strain is illustrated based on examples like *D. eschscholtzii* and *D. concentrica*. Additionally, 112 compounds have biological activities, such as anticancer, antiviral, antibacterial and antifungal activities, among others. The structures of secondary metabolites isolated from *Daldinia* sp. are also shown in Figure 2, Figure 3, Figure 4, Figure 5, Figure 6, Figure 7, Figure 8, Figure 9, Figure 10, Figure 11, Figure 12, Figure 13 and Figure 14. The structure type and bioactivity distribution of the compounds isolated from *Daldinia* sp. are also shown in Figure 15 and Figure 16.

The chemical structures of the 280 secondary metabolites from *Daldinia* species can mainly be classified into six types, including 21 terpenoids, 53 alkaloids, 125 polyketides, 58 polyphenols, 6 steroids and 17 other classes. However, among these 280 compounds, polyketides accounted for 44.64%, while terpenoids, alkaloids, polyphenols, steroids and other classes accounted for 7.50%, 18.93%, 20.72%, 2.14% and 6.07%, respectively (Figure 15).

As already mentioned in the previous sections, some strains of the *Daldinia* have already been targeted by chemical or molecular biology approaches. Interestingly, chemical and molecular biology methods, including chemical epigenetic modification, co-cultivation, OSMAC approach and genome mining, were employed to activate the silent/cryptic biosynthetic gene clusters within the genus *Daldinia.* Daldinsin (**85**) was isolated from *Daldinia eschscholzii* JC-15 based on the OSMAC strategy. Daldinones B (**146**) and E (**147**) were purified from the fungus *Daldinia* sp. treated with the epigenetic modifier SAHA. Dalestones A (**185**) and B (**186**) were obtained by the genomic mining of *Daldinia eschscholzii.* Eight new polyketides, daldinrin A–H (**107**–**114**), were isolated from the co-cultured fermentation products of *D. eschscholtzii* and *C. pseudomajus.* LC-MS/MS-based molecular networking has developed into a highly effective approach for the discovery of new natural products from complex mixtures. Six new tetramers (**205**–**210**), comprising the 5-methoxy-4-naphthol skeleton, were isolated from the fungus *Daldinia childiae* 047215 based on molecular networking analysis. The putative biosynthetic pathways of indolpolyketones A (**46**) and B (**47**), daldiconoid A (**74**), daldipyrenones A–C (**75**–**77**), daldiniaeschsones A (**78**) and B (**79**), (+)-orthosporin (**116**), nodulone (**171**), nodulones B (**197**) and C (**198**), (±) galewone (**213** and **214**) were also discussed. Daldinans B (**62**) and E (**64**) and entonalactam B (**67**) were synthesized.

Moreover, it is worth noting that nearly 40% (112 compounds) showed biological activities, including antimicrobial activities (24 compounds), cytotoxic activities (15 compounds), enzyme-inhibitory activities (17 compounds), antiviral activities (10 compounds), anti-inflammatory activities (15 compounds), antioxidant activities (16 compounds), anti-feedant activities (8 compounds) and other activities (7 compounds). Notably, antimicrobial (21.43%), enzyme-inhibitory (15.18%) and antioxidant activities (14.29%) represent the top three bioactivities (Figure 15 and Figure 16). It is important to highlight that 53 compounds exhibit potent activities. For example, a new indole alkaloid, (2*R*,4*S*)-indolchromin A (**49**), showed significant cytotoxic activity against the human breast cancer cell lines MDA-MB-231 with IC_50_ values of 27.9 nM, and against human breast cancer cell line MCF-7, with an IC_50_ value of 94.4 nM. Additionally, the samples were obtained from various environments: 39.64% from terrestrial plants, 24.64% from marine plants, 8.93% from animals and 26.79% from other resources.

In summary, species of *Daldinia* have been established as a significant reservoir of unique structures and a variety of secondary metabolites exhibiting a wide array of biological activities, thus uncovering their substantial, yet underutilized, potential in medicinal and agrochemical domains. Nonetheless, the scarcity of detailed pharmacological mechanisms hampers the application of most bioactive secondary metabolites. Therefore, future research should concentrate on elucidating pharmacological mechanisms, pharmacokinetics, medicinal chemistry, biosynthesis and other related areas to foster the advancement of innovative drug development.

**Table 1:** The producing strains, habitats and bioactivities of secondary metabolites from *Daldinia* species.

Compounds	Producing Strains	Habitats	Bioactivities	Refs
Methyl-7*α*-acetoxydeacetylbotryoloate (1)	*D. concentrica* (S0318)	Laojunshan, Yunnan Province, China	-	[14]
7*α*-Acetoxydeacetylbotryenedial (2)	*D. concentrica* (S0318)	Laojunshan, Yunnan Province, China	-	[14]
7*α*-Hydroxybotryenalol (3)	*D. concentrica* (S0318)	Laojunshan, Yunnan Province, China	-	[14]
7,8-Dehydronorbotryal (4)	*D. concentrica* (S0318)	Laojunshan, Yunnan Province, China	-	[14]
7*α*-Acetoxydehydrobotrydienal (5)	*D. concentrica* (S0318)	Laojunshan, Yunnan Province, China	-	[14]
7*α*-Acetoxy-15-methoxy-10-*O*-methyldeacetyldihydrobotrydial (6)	*D. concentrica* (S0318)	Laojunshan, Yunnan Province, China	-	[14]
7*α*-Hydroxy-10-*O*-methyldihydrobotrydial (7)	*D. concentrica* (S0318)	Laojunshan, Yunnan Province, China	-	[14]
7-Hydroxy-16-*O*-methyldeacetyldihydrobotrydial-hydrate (8)	*D. concentrica* (S0318)	Laojunshan, Yunnan Province, China	-	[14]
7-Hydroxydeacetyl-botryenalol (9)	*D. concentrica* (S0318)	Laojunshan, Yunnan Province, China	-	[14]
7*α*-Hydroxydihydrobotrydial (10)	*D. concentrica* (S0318)	Laojunshan, Yunnan Province, China	-	[14]
Rel-(1*S*,4*S*,5*R*,7*R*,10*R*)-10-desmethyl-1-methyl-11-eudesmene (11)	*D. childiae*	Lichen *Punctelia* sp., Guizhou Province, China	Inhibition rate (*α*-amylase) of 15.99% at the concentration of 1.5 mg/mL	[15]
Daldiconoids B–G (12–17)	*D. concentrica*	Fruiting bodies, Yunnan Province, China	Inhibition of IL-1*β*, IL-6 and TNF-*α* in LPS-stimulated RAW264.7 cells comparable to dexamethasone at a concentration of 10 μM	[16]
Concentricol (18)	*D. concentrica*	-	-	[17]
Concentricols B–D (19–21)	*D. concentrica*	*F. excelsior*, North Rhine Westphalia, Germany	-	[18]
(11)-Cytochalasa-6(12), 13,19-triene-1,21-dione-7,18-dihydroxy-16,18-dimethyl-l0-phenyl-(7S*,13*E*,16S*,18S*,19*E*) (22)	*Daldinia* sp.	*Quercus acutissima*, Tokushima	-	[19]
(11)-Cytochalasa-6(12),13-diene-1,21-dione-7,18-dihydroxy-16,18-dimethyl-l0-phenyl-(7S*,13*E*,16S*,18R*) (23)	*Daldinia* sp.	*Q. acutissima*, Tokushima	-	[19]
(11)-Cytochalasa-6(12),13-diene-1,21-dione-7,18,19-trihydroxy-16,18-dimethyl-l0-phenyl-(7S*,13*E*,16S*,18S*,19R*) (24)	*Daldinia* sp.	*Q. acutissima*, Tokushima	-	[19]
(11)-Cytochalasa-6(12),13-diene-1,21-dione-7,18-dihydroxy-16,18-dimethyl-19-methoxy-l0-phenyl-(7S*,13*E*,16S*,18S*,19R*) (25)	*Daldinia* sp.	*Q. acutissima*, Tokushima	-	[19]
(11)-Cytochalasa-6(12),13-diene-1,21-dione-7,18-dihydroxy-16,18-dimethyl-19-acetoxy-10-phenyl-(7S*,13*E*,16S*,18S*,19R*) (26)	*Daldinia* sp.	*Q. acutissima*, Tokushima	-	[19]
(11)-Cytochalasa-6(12),13,19-triene-l,21-dione-16,18-dimethyl-7-hydroxy-10-phenyl-(7S*,3*E*,16S*,18R*,19*E*) (27)	*Daldinia* sp.	*Q. acutissima*, Tokushima	-	[20]
(11)-Cytochalasa-6(12),13-diene-1,21-dione-16,18-dimethyl-7-hydroxy-10-phenyl-(7S*,13*E*,16S*,18S*) (28)	*Daldinia* sp.	*Q. acutissima*, Tokushima	-	[20]
(11)-Cytochalasa-6(12),13-diene-1,21-dione-7,19-dihydroxy-16,18-dimethyl-10-phenyl-(7S*,13*E*,16S*,18R*,19R*) (29)	*Daldinia* sp.	*Q. acutissima*, Tokushima	-	[20]
(11)-Cytochalasa-6,13,19-triene-l,21-dione-18-hydroxy-16,18-dimethyl-10-phenyl-(6*Z*,13*E*,16S*,18S*,19*E*) (30)	*Daldinia* sp.	*Q. acutissima*, Tokushima	-	[20]
(11)-Cytochalasa-6,13,19-triene-1,21-dione-12,18-dihydroxy-16,18-dimethyl-10-phenyl-(6*E*,13*E*,16S*,18S*,19*E*) (31)	*Daldinia* sp.	*Q. acutissima*, Tokushima	-	[20]
(11)-Cytochalasa-6,13,19-triene-l,21-dione-17-hydroxy-16,18-dimethyl-10-phenyl(6*Z*,13*E*,16S*,17*R*,18S*,19*E*) (32)	*Daldinia* sp.	*Q. acutissima*, Tokushima	-	[20]
(11)-Cytochalasa-13,19-diene-1,21-dione-6,7-epoxy-18-hydroxy-16,18-dimethyl-10-phenyl-(7S*,13*E*,16S*,18S*,19*E*) (33)	*Daldinia* sp.	*Q. acutissima*, Tokushima	-	[20]
(11)-Cytochalas-13-ene-1,21-dione-6,7-epoxy-18,19-dihydroxy-16,18-dimethyl-l0-phenyl-(7S*,13*E*,16S*,18S*,19R*) (34)	*Daldinia* sp.	*Q. acutissima*, Tokushima	-	[20]
(11)-Cytochalasa-6(12),13,19-triene-1,17,21-trione-7,18-dihydroxy-16,18-dimethyl-10-phenyl-(7S*,13*E*,16S*,l8R*,19*E*) (35)	*Daldinia* sp.	*Q. acutissima*, Tokushima	-	[20]
(11)-Cytochalasa-5,13-19-triene-1,21-dione-7-hydroperoxy-17-hydroxy-16,18-dimethyl-10-phenyl-(5Z*,7S*,13*E*, 16S*,17R*,18S*,19*E*) (36)	*Daldinia* sp.	*Q. acutissima*, Tokushima	-	[20]
22-Oxa-(12)-cytochalasa-6(12),13,19-triene-l,21-dione-7,18-dihydroxy-16,18-dimethyl-10-phenyl-(7S*,13*E*,16S*,18S*,19*E*) (37)	*Daldinia* sp.	*Q. acutissima*, Tokushima	-	[20]
[11]-Cytochalasa-6(12),13-diene-1,21-dione-7,19-dihydroxy-16,18-dimethyl-10-phenyl-(7S*,13*E*,16S*,18R*,19R*) (38)	*D. vernicosa*	*Q. acutissima*, Tokushima	-	[21]
Daldinin (39)	*D. concentrica*	Pumat National Park of Nghe An Province, Vietnam	IC_50_ (cytotoxicity) 11.4–13.5 µM	[22]
[11]-Cytochalasa-6(12),13-diene-1,21-dione-7,18,19-trihydroxy-16,18-dimethyl-10-phenyl-(7S*,13*E*,16S*,18S*,19R*) (40)	*D. concentrica*	Pumat National Park of Nghe An Province, Vietnam	IC_50_ (cytotoxicity) 23.0–58.2 µM	[22]
[11]-Cytochalasa-6(12),13diene-1,21 dione-7,18-dihydroxy-16,18-dimethyl-10-phenyl-(7S*,13*E*,16S*,18R*) (41)	*D. concentrica*	Pumat National Park of Nghe An Province, Vietnam	IC_50_ (cytotoxicity) 23.0–58.2 µM	[22]
[11]-Cytochalasa-5(6),13-diene-1,21-dione-7,18-dihydroxy-16,18-dimethyl-10-phenyl-(7S*,13*E*,16S*,18R*) (42)	*D. eschscholtzii* HJ001	*Bruguiera sexangula var. rhynchopetala*, South China Sea, China	MIC (antibacterial) 50 µg/mL	[23]
21-(Acetyloxy)-6,13,14-trihydroxy-16,18-dimethyl-10-phenyl[11]cytochalasa-7,19-dien-1-one (43)	*D. concentrica*	Laojunshan, Yunnan Province, China	-	[24]
Phenochalasin B (44)	*D. concentrica*	*F. excelsior*, North Rhine Westphalia, Germany	-	[25]
Cytochalasin O (45)	*D. eschscholzii* MCZ-18	*C. tagal*, Hainan Province, China	-	[26]
Indolpolyketones A (46) and B (47)	*D. escosholzii*	*T. aridifolia*	IC_50_ (antiviral) 45.2, 31.4 µM	[27]
(2*S*,4*R*)-Indolchromin A (48)	*D. eschscholzii*	*T. aridifolia*	MIC (antibacterial) 2.1–6.4 µM	[28]
(2*R*,4*S*)-Indolchromin A (49)	*D. eschscholzii*	*T. aridifolia*	MIC (antibacterial) 1.3–6.2 µM, IC_50_ (cytotoxicity) 27.9–94.4 nM	[28]
(2*S*,4*S*)-Indolchromin A (50)	*D. eschscholzii*	*T. aridifolia*	-	[28]
(2*R*,4*R*)-Indolchromin A (51)	*D. eschscholzii*	*T. aridifolia*	-	[28]
(2*S*,4*R*)-Indolchromin B (52)	*D. eschscholzii*	*T. aridifolia*	-	[28]
(2*R*,4*S*)-Indolchromin B (53)	*D. eschscholzii*	*T. aridifolia*	MIC (antibacterial) 6.2–6.7 µM	[28]
(2*S*,4*S*)-Indolchromin B (54)	*D. eschscholzii*	*T. aridifolia*	IC_50_ (cytotoxicity) 131.2 nM	[28]
(2*R*,4*R*)-Indolchromin B (55)	*D. eschscholzii*	*T. aridifolia*	-	[28]
Dalesindoloids A–B (56–57)	*D. eschscholzii*	*T. aridifolia*	IC_50_ (cytotoxicity) 1.0–7.4 µM	[29]
3-(1*H*-indole-3ylmethyl)-2-oxindole (58)	*D. eschscholzii*	*T. aridifolia*	-	[29]
Daldinans B–C (59–60)	*D. concentrica*	Yamanashi Prefecture, Japan	-	[30]
Daldinans A–B (61–62)	*D. concentrica*	Odaesan National Park, Korea	IC_50_ (antioxidant) 12.62–39.67 µM	[31]
Daldinans D–G (63–66)	*D. concentrica*	Odaesan National Park, Korea	IC_50_ (antioxidant) 2.65–3.50 µM	[31]
Entonalactam B (67)	*Daldinia* sp.	-	-	[32]
(±) Childinins C (68)	*D. childiae*	Kunming Botanical Garden, China	-	[33]
Daldinin A (69)	*D. concentrica*	Muju county, Jeonbuk province, Korea	IC_50_ (radical scavenging) 10.4 μM	[34]
1-(3-Indolyl)-2*R*,3-dihydroxypropan-1-one (70)	*D. eschschilzii*	*Scaevola serica*, Hainan Province, China	-	[35]
3-Ethyl-2,5-pyrazinedipropanoic acid (71)	*D. eschschilzii*	*S. serica*, Hainan Province, China	-	[35]
2,5-Pyrazinedipropanoic acid (72)	*D. eschschilzii*	*S. serica*, Hainan Province, China	-	[35]
Cyclo-(Phe-Tyr) (73)	*D. eschschilzii*	*S. serica*, Hainan Province, China	-	[35]
Daldiconoid A (74)	*D. concentrica*	Fruiting bodies, Yunnan Province, China	Inhibition of IL-1*β*, IL-6 and TNF-*α* in LPS stimulated RAW264.7 cells comparable to those of dexamethasone at a concentration of 10 μM	[16]
Daldipyrenones A–C (75–77)	*D. pyrenaica* 047188	Lichen *Myelochroa aurulenta*, Korea	EC_50_ (antimelanogenic) 3.36 mM	[36]
Daldiniaeschsones A (78) and B (79)	*D. eschscholtzii* SDBRCMUNKC745	*Cinnamomum bejolghota*, Chiang Mai, Thailand	IC_50_ (*α*-glucosidase inhibitory) 0.16–0.23 mM	[37]
Nodulisporin G (80)	*D. eschscholzii*	*T. aridifolia*, Nanjing, China	-	[38]
8-*O*-methylnodulisporin F (81)	*D. eschscholtzii* HJ004	*B. sexangula*, South China Sea, China	MIC (antibacterial) 6.22–12.5 µg/mL	[39]
Nodulisporin H (82)	*D. eschscholtzii* HJ004	*B. sexangula*, South China Sea, China	MIC (antibacterial) 6.22–12.5 µg/mL	[39]
Daldiniside A (83)	*D. eschschilzii*	*S. serica*, Hainan Province, China	-	[35]
7-*O*-*α*-*D*-ribosyl-5-hydroxy-2-propylchromone (84)	*D. eschscholtzii* J11	*E. pekinensis*, Yunnan Province, China	-	[40]
Daldinsin (85)	*D. eschscholzii* JC-15	*D. officinale*, Yunnan, Province, China	Inhibition rate (acetylcholinesterase) of 38.8% at the concentration of 50 µM	[41]
Karimanone (86)	*D. eschscholtzii* KJMT FP4.1	*Xestospongia* sp., Karimonjawa National Park	MIC (antibacterial) 125 µg/mL	[42]
(*S*)-5-Hydroxy-2-methyl-4-chromanone (87)	*D. eschscholtzii* KJMT FP4.1	*Xestospongia* sp., Karimonjawa National Park	MIC (antibacterial) 62.5 µg/mL	[42]
5,7-Dihydroxy-2-methyl-4-chromanone (88)	*D. eschscholtzii* KJMT FP4.1	*Xestospongia* sp., Karimonjawa National Park	MIC (antibacterial) 125 µg/mL	[42]
(*R*)-5-hydroxy-8-methoxy-2-methylchroman-4-one (89)	*D. eschscholtzii*	Broad-leaved tree, Deyang City, Sichuan Province, China	-	[43]
7-Hydroxy-5-methoxy-2,3-dimethylchromone (90)	*D. eschscholtzii* HJ004	*B. sexangula* var. *rhynchopetala*, Hainan Province, China	IC_50_ (*α*-glucosidase inhibitory) 13.0 µM	[44]
5-Methoxy-2-propanone (91)	*D. eschscholtzii* HJ004	*B. sexangula* var. *rhynchopetala*, Hainan Province, China	-	[44]
7-Ethyl-8-hydroxy-6-methoxy-2,3-dimethylchromone (92)	*D. eschscholtzii* HJ004	*B. sexangula* var. *rhynchopetala*, Hainan Province, China	-	[44]
2,3-Dihydro-5-methoxy-2-methylchromen-4-one (93)	*D. eschscholtzii* HJ004	*B. sexangula* var. *rhynchopetala*, Hainan Province, China	IC_50_ (*α*-glucosidase inhibitory) 15.0 µM	[44]
8-Methoxy-2-methyl-4*H*-1-benzopyran-4-one (94)	*D. eschscholtzii* HJ004	*B. sexangula* var. *rhynchopetala*, Hainan Province, China	-	[44]
7-Hydroxy-2,5-dimethyl-4*H*-1-benzopyran-4-one (95)	*D. eschscholtzii* HJ004	*B. sexangula* var. *rhynchopetala*, Hainan Province, China	-	[44]
(5*R*,7*R*)-5,7-Dihydroxy-2-methyl-5,6,7,8-tetrahydro-4*H*-chromen-4-one (96)	*Daldinia* sp. CPCC 400770	Laojunshan, Dali Bai Autonomous Region, Yunnan Province, China	IC_50_ (antiviral) 16.1 µM	[45]
(5*R*,7*R*)-5,7-Dihydroxy-2-copy-5,6,7,8-tetrahydro-4*H*-chromen-4-one (97)	*Daldinia* sp. CPCC 400770	Laojunshan, Dali Bai Autonomous Region, Yunnan Province, China	-	[45]
(5*R*,7*R*)-5,7-Dihydroxy-2-methyl-5,6,7,8-tetrahydro-4*H*-chromen-4-one (98)	*Daldinia* sp. CPCC 400770	Laojunshan, Dali Bai Autonomous Region, Yunnan Province, China	IC_50_ (antiviral) 9.0 µM	[45]
(5*R*,7*S*)-5,7-dihydroxy-2-methyl-5,6,7,8-tetrahydro-4*H*-chromen-4-one (99)	*Daldinia* sp. CPCC 400770	Laojunshan, Dali Bai Autonomous Region, Yunnan Province, China	-	[45]
8-Ethyl-7-hydroxy-5-carboxyl-2,3-dimethylchromone (100)	*D. eschscholtzii* HJ004	*B. sexangula*, South China Sea, China	IC_50_ (antioxidant) 195.03 µM	[46]
5-Hydroxy-2-methyl-4*H*-chromen-4-one (101)	*D. eschscholtzii* HJ004	*B. sexangula*, South China Sea, China	IC_50_ (antioxidant) 20.79 µM	[46]
5,8-Dihydroxy-2-methylchromone (102)	*D. eschscholtzii* HJ004	*B. sexangula*, South China Sea, China	IC_50_ (antioxidant) 8.90 µM	[46]
5,7-Dihydroxy-2-propylchromone (103)	*D. eschscholtzii* HJ004	*B. sexangula*, South China Sea, China	IC_50_ (antioxidant) 5.57 µM	[46]
3,5-Dihydroxy-2-methyl-4*H*-chromen-4-one (104)	* D. eschscholtzii * A630	* P. cablin * , Guangdong Province, China	-	[47]
(2*R*,4*R*)-3,4-dihydro-5-methoxy-2-methyl-2*H*-1-benzopyran-4-ol (105)	*D. eschschiltzii* KBJYZ-1	*P. indica*, Guangdong Province, China	-	[48]
5-Hydroxy-8-methoxy-2-methyl-4*H*-1-benzopyran-4-one (106)	*D. eschscholzii* MCZ-18	*C. tagal*, Hainan Province, China	-	[26]
Daldinrins A–H (107–114)	*D. eschscholtzii*	*Colletotrichum sinensis*, Yunnan Province, China	MIC (antibacterial) 1.0 µg/mL	[49]
(+)-Mellein (115)	*D. eschscholtzii* SDBRCMUNKC745	*C. bejolghota,* Chiang Mai, Thailand	IC_50_ (*α*-glucosidase inhibitory) 0.76 mM	[37]
(+)-Orthosporin (116)	* D. eschscholtzii *	* P. exul * , Chiang Mai, Thailand	CC_50_ (cytotoxicity) 89.0 µM	[50]
(3*R*)-Methyl-6,8-dihydroxy-7-methyl-3,4-dihydroisocoumarin (117)	*D. eschscholtzii* J11	*E. pekinensis*, Yunnan Province, China	-	[40]
6-Hydroxymellein (118)	*D. eschscholtzii* J11	*E. pekinensis*, Yunnan Province, China	-	[40]
2-Acetyl-7-methoxybenzofuran (119)	*D. eschscholzii* MCZ-18	*C. tagal*, Hainan Province, China	MIC (antibacterial) 12.5–25 µg/mL	[26]
4,8-Dimethoxy-1*H*-isochromen-1-one (120)	*D. eschscholzii* MCZ-18	*C. tagal*, Hainan Province, China	-	[26]
(+)-Citreoisocoumarin (121)	*D. eschscholzii* MCZ-18	*C. tagal*, Hainan Province, China	-	[26]
Daldinisides B–C (122–123)	*D. eschschilzii*	*S. serica*, Hainan Province, China	-	[35]
4-Methoxy-5-carboxymethoxy-6-pentyl-2*H*-pyran-2-one (124)	*D. concentrica*	European ash, Germany	-	[25]
Childinins A (125), F–G (126–127)	*D. childiae*	Kunming, Yunnan Province, China	-	[33]
Daldineschscin B (128)	*D. eschscholtzii* J11	*E. pekinensis,* Yunnan Province, China	-	[40]
Helicascolides D (129) and E (130)	*D. eschscholtzii* HJ004	*B. sexangula,* Hainan Province, China	-	[44]
Helicascolides A (131) and B (132)	*D. eschscholtzii* HJ004	*B. sexangula,* Hainan Province, China	IC_50_ (*α*-glucosidase inhibitory) 16 μM	[44]
Helicascolide G (133)	*D. eschscholtzii* HJ004	*B. sexangula,* South China Sea, China	-	[46]
Helicascolide A (134)	*D. eschscholzii*	*Gracilaria* sp., South Sulawesi Island, Indonesia	Against the plant pathogenic fungus	[51]
Daldinolides A–B (135–136)	*D. concentrica*	Yamanashi Prefecture, Japan	-	[30]
Concentricolide (137)	*D. concentrica*	Yunnan Province, China	EC_50_ (inhibited HIV-1) 0.31 µg/mL, EC_50_ (exhibited the blockage) 0.83 µg/mL	[52]
Daldinins A–B (138–139)	*D. concentrica*	Yunnan Province, China	-	[53]
4-Isoochracein (140)	*D. concentrica*	Yunnan Province, China	-	[53]
Hydroxyisoochracein (141)	*D. concentrica*	Yunnan Province, China	-	[53]
Curuilignan D (142)	*D. concentrica*	European ash, North Rhine Westphalia, Germany	-	[25]
Daldinione E (143)	*D. eschscholtzii* HJ004	*B. sexangula,* South China Sea, China	-	[44]
(*E*)-6-(non3-en-1-yl)-2*H*-pyran-2-one (144)	*D. eschscholtzii*	Broad-leaved tree, Sichuan Province, China	-	[43]
Daldinone A (145)	*D. concentrica*	European ash, North Rhine Westphalia, Germany	-	[25]
Daldinone B (146)	*Daldinia* sp.	*C. cainito*	IC_50_ (radical scavenging) 3.1 μM	[54]
Daldinone E (147)	*Daldinia* sp.	*C. cainito*	IC_50_ (radical scavenging) 3.6 μM	[54]
Daldinones F–G (148–149)	*D. eschscholtzii*	* P. exul * , Chiang Mai, Thailand	-	[50]
Daldinones C–D (150–151)	*D. eschscholtzii*	* P. exul * , Chiang Mai, Thailand	IC_50_ (cytotoxicity) 41.0–49.5 µM	[50]
Dalmanol C (152)	*D. eschscholzii*	*Tenodera aridifolia*, Nanjing, China	-	[38]
Daldinone F (153)	*D. eschscholzii*	*T. aridifolia*, Nanjing, China	IC_50_ (cytotoxicity) 9.59 µM	[38]
3,4,5-Trihydroxy-1-tetralone (154)	*D. concentrica*	European ash, North Rhine Westphalia, Germany	-	[25]
(1*R*,4*R*)-5-methoxy-1,2,3,4-tetrahydronaphthalene-1,4-dio (155)	*D. eschschiltzii* KBJYZ-1	*P. indica*, Guangdong Province, China	-	[48]
(+)-Regiolone (isosclerone) (156)	*D. eschscholtzii* SDBRCMUNKC745	*C. bejolghota*, Chiang Mai, Thailand	IC_50_ (*α*-glucosidase inhibitory) 0.85 mM	[37]
(+)-3,4-Dihydro-3,4,8-trihydroxy-1(2*H*)-naphthalenone (157)	*D. eschscholtzii* SDBRCMUNKC745	*C. bejolghota*, Chiang Mai, Thailand	IC_50_ (*α*-glucosidase inhibitory) 0.55 mM	[37]
Daldiniones C–D (158–159)	*D. eschscholtzii* HJ004	*B. sexangula*, the South China Sea, China	-	[44]
4*R*-(*O*)-methylsclerone (160)	*D. eschscholtzii* HJ004	*B. sexangula*, the South China Sea, China	-	[44]
(-)-Cis-(3R*,4S*)-3,4,8-trihydroxy-6,7-dimethyl-3,4-dihydronaphthalen-1(2*H*)-one (161)	*D. eschscholtzii* HJ004	*B. sexangula*, the South China Sea, China	IC_50_ (*α*-glucosidase inhibitory) 21 μM	[44]
(4R*)-4,8-dihydroxy-3-hydro-5-methoxy-1-naphthhthalenone (162)	*Daldinia* sp.	*C. tagal*, Hainan Province, China; oak leaves, Tokushima	-	[21,26]
4,6,8-Trihydroxide-3,4-dihydronaphthhalen-1 (2*H*)-one (163)	*D. eschscholzii*	*S. sericea*, Hainan Province, China	-	[35]
(-)-Regiolone (164)	*D. childiae*	Lichen *Punctelia* sp, Guizhou Province, China	-	[15]
(1*R*, 4*R*)-5-methoxy-1,2,3,4-tetrahydronaphthalene-1,4-dio (165)	*D. eschschiltzii* KBJYZ-1	* P. indica * , Guangdong Province, China	-	[48]
Daldinrin K (166)	*D. eschscholtzii*	*C. sinensis*, Yunnan Province, China	Anti-feedant activities with feeding deterrence index of 81%	[49]
Dalesconoside F (167)	*D. eschscholzii* MCZ-18	*C. tagal*, Hainan Province, China	-	[26]
Nodulisporone (168)	*D. eschscholzii* MCZ-18	*C. tagal*, Hainan Province, China	-	[26]
Xylariol A (169)	*D. eschscholzii* MCZ-18	*C. tagal*, Hainan Province, China	-	[26]
(4*R*)-3,4-dihydro-4,5-dihydroxynaphthalen-1(2*H*)-one (170)	*D. eschscholzii* MCZ-18	*C. tagal*, Hainan Province, China	MIC (antimicrobial) 25–50 µg/mL	[26]
Nodulone (171)	* D. eschscholtzii *	* P. exul * , Chiang Mai, Thailand	-	[50]
Daldiquinone (172)	*D. concentrica*	Yamanashi Prefecture, Japan	IC_50_ (antiangiogenesis activity) 7.5 µM	[30]
Dalditene A (173)	*D. eschschiltzii* KBJYZ-1	* P. indica * ,Guangdong Province, China	IC_50_ (anti-inflammatory) 12.9 µM	[48]
Dalesconoside D (174)	*D. eschscholzii* MCZ-18	*C. tagal*, Hainan Province, China	-	[26]
Dalesconoside E (175)	*D. eschscholzii* MCZ-18	*C. tagal*, Hainan Province, China	-	[26]
5-Hydroxy-2-methoxy-6,7-dimethyl-1,4-naphthoquinone (176)	*D. eschscholtzii* HJ004	*B. sexangula*, South China Sea, China	MIC (antibacterial) 12.5 µg/mL	[39]
5-Hydroxy-2-methoxynaphtho[9–*c*]furan-1,4-dione (177)	*D. eschscholtzii* HJ004	*B. sexangula*, South China Sea, China	IC_50_ (*α*-glucosidase inhibitory) 5.7 µg/mL	[39]
3-Alkyl-5-methoxy-2-methyl-1,4-benzoquinones,3-heneicosyl (178),	*D. concentrica*	Laojunshan, Yunnan Province, China	-	[55]
3-Docosyl-5-methoxy-2-methyl-1,4-benzoquinone (179)	*D. concentrica*	Laojunshan, Yunnan Province, China	-	[55]
5-Methoxy-2-methyl-3-tricosyl-1,4-benzoquinone (180)	*D. concentrica*	Laojunshan, Yunnan Province, China	-	[55]
Daldinrin J (181)	*D. eschscholtzii*	*C. sinensis*, Yunnan Province, China	Anti-feedant activities with feeding deterrence index of 81%	[49]
2-Acetyl-7-methoxybenzofuran (182)	*D. eschscholtzii* HJ004	*B. sexangula*, South China Sea, China	IC_50_ (*α*-glucosidase inhibitory) 1.1 µg/mL	[39]
Daldisones A–B (183–184)	*Daldinia* sp. CPCC 400770	-	IC_50_ (antiviral) 16.0–7.4 µM, MIC (antibacterial) 16.0–32 µg/mL	[56]
Dalestones A–B (185–186)	*D. eschscholzii*	-	Inhibition of IL-1*β*, IL-6 and TNF-*α* in LPS stimulated RAW264.7 cells comparable to those of dexamethasone at a concentration of 20 μM	[57]
Daldineschscin A (187)	*D. eschscholtzii* J11	*E. pekinensis*, Yunnan Province, China	-	[40]
Xylariphilone (188)	*D. eschscholtzii* J11	*E. pekinensis*, Yunnan Province, China	IC_50_ (radical scavenging) 49.13 μM	[40]
Dalesconoside B (189)	*D. eschscholzii* MCZ-18	*C. tagal*, Hainan Province, China	-	[26]
Fusaraisochromenone (190)	*D. eschscholzii* MCZ-18	*C. tagal*, Hainan Province, China	MIC (antibacterial) 6.25–25 µg/mL	[26]
Childinin B (191)	*D. childiae*	Kunming Botanical Garden, China	MIC_90_ (antibacterial) 54.9 µg/mL	[33]
Daldiniones A–B (192–193)	*D. eschscholtzii* HJ004	*B. sexangula*, Hainan Province, China	-	[44]
Eschscholin A (194)	* D. eschscholtzii * A630	*P. cablin*, Guangdong Province, China	-	[47]
3-Ene-2-methyl-2*H*-1-benzopyran-5-ol (195)	* D. eschscholtzii * A630	*P. cablin*, Guangdong Province, China	-	[47]
Daldionin (196)	* D. eschscholtzii *	* P. exul * , Chiang Mai, Thailand	Antiproliferative activity against HUVEC (GI_50_ 98.4 μM) and K-562 cell lines (GI_50_ 85.5 μM)	[50]
Nodulones B–C (197–198)	* D. eschscholtzii *	* P. exul * , Chiang Mai, Thailand	-	[50]
Pyranlyketide (199)	*D. eschscholtzii*	*C. sinensis*, Yunnan Province, China	-	[49]
1,1′,3′,3″-Ternaphthalene-5,5′,5″-trimethoxy-4,4′,4″-triol (200)	*D. childiae* 047215	Lichen *P. adaugescens*, South Korea	-	[58]
3,1′,3′,3″-Ternaphthalene-5,5′,5″-trimethoxy-4,4′,4″-triol (201)	*D. childiae* 047215	Lichen *P. adaugescens*, South Korea	IC_50_ (adiponectin synthesis-promoting activity) 30.8 μM	[58]
1,1′,3′,1″-Ternaphthalene-5,5′,5″-trimethoxy-4,4′,4″-triol (202)	*D. childiae* 047215	Lichen *P. adaugescens*, South Korea	-	[58]
3,1′,3′,1″-Ternaphthalene-5,5′,5″-trimethoxy-4,4′,4″-triol (203)	*D. childiae* 047215	Lichen *P. adaugescens*, South Korea	-	[58]
Nodulisporin A (204)	*D. childiae* 047215	Lichen *P. adaugescens*, South Korea	IC_50_ (adiponectin synthesis-promoting activity) 15.2 μM	[58]
1,1′,3′,3″,1″,1‴-Quaternaphthalene-5,5′,5″,5‴-tetramethoxy-4,4′,4″4‴-tetraol (205)	*D.childiae* 047219	Lichen *P. adaugescens*, Hannah Mountain in Jeju Island, South Korea	-	[59]
1,1′,3′,1″,3″,3‴-Quaternaphthalene-5,5′,5″,5‴-tetramethoxy-4,4′,4″4‴-tetraol (206)	*D. childiae* 047219	Lichen *P. adaugescens*, Hannah Mountain in Jeju Island, South Korea	-	[59]
3,3′,1′,1″,3″,3‴-Quaternaphthalene-5,5′,5″,5‴-tetramethoxy-4,4′,4″4‴-tetraol (207)	*D. childiae* 047219	Lichen *P. adaugescens*, Hannah Mountain in Jeju Island, South Korea	-	[59]
1,1′,3′,3″,1″,3‴-Quaternaphthalene-5,5′,5″,5‴-tetramethoxy-4,4′,4″4‴-tetraol (208)	*D. childiae* 047219	Lichen *P. adaugescens*, Hannah Mountain in Jeju Island, South Korea	-	[59]
3,1′,3′,1″,3″,3‴-Quaternaphthalene-5,5′,5″,5‴-tetramethoxy-4,4′,4″4‴-tetraol (209)	*D. childiae* 047219	Lichen *P. adaugescens*, Hannah Mountain in Jeju Island, South Korea	-	[59]
3,1′,3′,3″,1″,3‴-Quaternaphthalene-5,5′,5″,5‴-tetramethoxy-4,4′,4″4‴-tetraol (210)	*D. childiae* 047219	Lichen *P. adaugescens*, Hannah Mountain in Jeju Island, South Korea	-	[59]
Nodulisporins B (211) and E (212)	*D. childiae* 047219	Lichen *P. adaugescens*, Hannah Mountain in Jeju Island, South Korea	Inhibition rate (NO on LPS-induced RAW cells) of 50% at the concentration of 25–50 µM	[59]
(±)-Galewone (213–214)	*D. eschscholzii* IFB-TL01	*T. aridifolia*	IC_50_ (reduced cell viability) 3.73–10.10 μM	[60]
(±)-Spirodalesol (215–216)	*D. eschscholzii*	*T. aridifolia*	IC_50_ (suppressed secretion of IL-1*β*) 0.67–0.70 μM	[61]
Selesconol (217)	*D. eschscholzii* IFB-TL01	*T. aridifolia*	-	[62]
(±)-Dalesconol A (218–219)	*D. eschscholzii* IFB-TL01	*T. aridifolia*	-	[63]
(±)-Dalesconol B (220–221)	*D. eschscholzii* IFB-TL01	*T. aridifolia*	-	[63]
Dalesconosides A and C (222–223)	*D. eschscholzii* MCZ-18	*C. tagal*, Hainan Province, China	MIC (antibacterial) 12.5–25 µg/mL	[26]
8-Methoxynaphthalen-1-ol (224)	*D. loculata*	Lichen, Laojunshan, Yunnan Province, China	-	[64]
Daldiniol G (225)	*Daldinia* sp. TJ403-LS1	*A. roxburghi*, Hubei Province, China	IC_50_ (BChE inhibitory activity) 15.53 μM	[65]
1,8-Dimethoxynaphthalene (226)	*D. eschscholtzii* J11	*E. pekinensis*, Yunnan Province, China	-	[40]
1,3,8-Trimethoxynaphtho[9–*c*]furan (227)	*D. eschscholtzii* HJ004	*B. sexangula*, South China Sea, China	-	[39]
4:5:4′:5′-Tetrahydroxy-1:1′-binaphthyl (228)	*D. concentrica*	European ash, North Rhine Westphalia, Germany	-	[25]
Daldiniols A–D (229–232)	*Daldinia* sp. TJ403-LS1	*A. roxburghi*, Hubei Province, China	IC_50_ (BChE inhibitory activity) 6.93 μM	[65]
4-Hydroxy-3-(3-methylbut-3-en-l-ynyl) benzyl alcohol (233)	*Daldinia* sp. TJ403-LS1	*A. roxburghi*, Hubei Province, China	-	[65]
4-Methoxy-3-(3-methylcut-3-en-l-ynyl) benzyl alcohol (234)	*Daldinia* sp. TJ403-LS1	*A. roxburghi*, Hubei Province, China	IC_50_ (BChE inhibitory activity) 16.00 μM	[65]
Daldiniol E (235)	*Daldinia* sp. TJ403-LS1	*A. roxburghi*, Wuhan, Hubei Province, China	-	[65]
Dalditone B (236)	*D. eschschiltzii* KBJYZ-1	* P. indica * , Guangdong Province, China	-	[48]
Orcinotriol (237)	*D. eschschilzii*	*S. serica*, Hainan Province, China	-	[35]
4-(2-Hydroxyethyl) phenol (238)	*D. loculata*	Laojunshan, Yunnan Province, China	-	[65]
Daldins A–C (239–241)	*D. concentrateca* S0318	Laojunshan, Yunnan Province, China	-	[66]
2-Hydroxymethyl-3-(1-hydroxypropyl) phenol (242)	*D. concentrateca* S0318	Laojunshan, Yunnan Province, China	-	[66]
Daldinrin L (243)	*D. eschscholtzii*	*C. sinensis*, Yunnan Province, China	Anti-feedant activity with feeding deterrence index of 70% at concentrations of 50 μg/cm^2^	[49]
Stachyline C (244)	*Daldinia* sp. CPCC 400770	Laojunshan, Yunnan Province, China	-	[45]
3-Methoxy-4-hydroxyphenylethanol (245)	*Daldinia* sp. CPCC 400770	Laojunshan, Yunnan Province, China	-	[45]
3-Hydroxy-4-methoxy-phenylethanol (246)	*Daldinia* sp. CPCC 400770	Laojunshan, Yunnan Province, China	-	[45]
Childinin D (247)	*D. childiae*	Kunming Botanical Garden, China	-	[33]
Childinin E (248)	*D. childiae*	Kunming Botanical Garden, China	-	[33]
2-(Hydroxymethyl)-3-(1-hydroxypropyl) phenol (249)	*D. concentrica*	Laojunshan, Yunnan Province, China	-	[53]
AB5046A (250)	*D. eschscholtzii* KJMT FP4.1	*Xestospongia* sp., Karimonjawa National Park	MIC (antibacterial) 125 µg/mL	[42]
(3*S*)-3,8-dihydroxy-6,7-dimethyl-*α*-tetralone (251)	*D. eschscholtzii* PSU-STD57	*B. gymnorrhiza*, Suratthani province, Thailand	-	[67]
3-Hydroxy-1-(2,6-dihydroxyphenyl)butan-1-one (252)	*D. eschscholtzii* J11	*E. pekinensis*, Yunnan Province, China	IC_50_ (radical scavenging) 43.66 μM	[40]
1-(2,6-Dihydroxy-phenyl)butan-1-one (253)	*D. eschscholtzii* J11	*E. pekinensis*, Yunnan Province, China	IC_50_ (radical scavenging) 28.08 μM	[40]
1-(2,6-Dihydroxyphenyl) ethan-1-one (254)	*D. eschscholtzii* J11	*E. pekinensis*, Yunnan Province, China	-	[40]
Daldispols A–C (255–257)	*Daldinia* sp. CPCC 400770	Laojunshan, Yunnan Province, China	IC_50_ (antiviral) 12.7–6.4 µM	[45]
Ergosterol (258)	*D. concentrica*	Pumat National Park of Nghe An Province, Vietnam	IC_50_ (cytotoxicity) 21.5–43.6 µM	[22]
Ergosterol peroxide (259)	*D. concentrica*	Pumat National Park of Nghe An Province, Vietnam	IC_50_ (cytotoxicity) 46.9–35.2 µM	[22]
Childinasterone A (260)	*D. childiae*	Kunming Botanical Garden, China	IC_50_ (anti-NO activity) 21.2 µM	[33]
Daldinialone (22*R*-hydroxylanosta-7,9 (11), 24-trien-3-one) (261)	*D. concentrica*	European ash, North Rhine Westphalia, Germany	-	[25]
22*E*-ergosta-4,6,8(14),22-tetraen-3-one (262)	*D. eschscholtzii* J11	*E. pekinensis*, Yunnan Province, China	-	[40]
Dankasterone A (263)	*D. eschscholzii* MCZ-18	*C. tagal*, Hainan Province, China	-	[26]
1-Isopropy-2,7-dimethylnaphthhalene (264)	*D. concentrica*	Laojunshan, Yunnan Province, China	-	[24]
Coldols A–C (265–267)	*D. eschscholtzii*	*C. sinensis*, Yunnan Province, China	-	[49]
Coldiol (268)	*D. eschscholtzii*	*C. sinensis*, Yunnan Province, China	Anti-feedant activity with feeding deterrence index of 20% at concentrations of 50 μg/cm^2^	[49]
Daldinrin I (269)	*D. eschscholtzii*	*C. sinensis*, Yunnan Province, China	Anti-feedant activity with feeding deterrence index of 97% at concentrations of 50 μg/cm^2^	[49]
(2*S*,4*S*,5*R*)-hept-6-en-2,4,5-triol (270)	*D. eschscholtzii*	*C. sinensis*, Yunnan Province, China	Anti-feedant activities with feeding deterrence index of 30% at concentrations of 50 μg/cm^2^	[49]
Hypoxylonol H (271)	*D. eschscholtzii*	*C. sinensis*, Yunnan Province, China	Anti-feedant activities with feeding deterrence index of 50% at concentrations of 50 μg/cm^2^	[49]
5-Methyl-2-vinyltetrahydrofuran-3-ol (272)	*D. eschscholtzii*	*C. sinensis*, Yunnan Province, China	Anti-feedant activities with feeding deterrence index of 15% at concentrations of 50 μg/cm^2^	[49]
2-Phenylethyl-*β*-*D*-glucopyranoside (273)	*Daldinia* sp. TJ403-LS1, *D. eschscholtzii* HJ004	*A. roxburghi*, Hubei Province, *B. sexangula*, South China Sea, China	IC_50_ (BChE inhibitory activity) 23.33 μM	[46,65]
Daldiniol F (274)	*Daldinia* sp. CPCC 400770	Laojunshan, Yunnan Province, China	IC_50_ (antiviral) 12.5 µM	[45]
2,4,5-Heptanetriol (275)	*D. childiae*	Lichen *Punctelia* sp, Guizhou Province, China	-	[15]
6-Heptene-2,4,5-triol (276)	*D. childiae*	Lichen *Punctelia* sp., Guizhou Province, China	-	[15]
Eschschilin B (277)	*D. eschschiltzii* KBJYZ-1	* P. indica * , Guangdong Province, China	IC_50_ (anti-inflammatory) 19.3 µM	[48]
Dalditone A (278)	*D. eschschiltzii* KBJYZ-1	* P. indica * , Guangdong Province, China	-	[48]
Eschscholin A (279)	*D. eschschiltzii* KBJYZ-1	* P. indica * , Guangdong Province, China	-	[48]
Daldinin C (280)	*D. concentrica*	Laojunshan, Yunnan Province, China	-	[53]
